# Electric Field of DNA in Solution: Who Is in Charge?

**DOI:** 10.1103/physrevx.14.031042

**Published:** 2024-09-05

**Authors:** Jonathan G. Hedley, Kush Coshic, Aleksei Aksimentiev, Alexei A. Kornyshev

**Affiliations:** Department of Chemistry, Imperial College London, Molecular Sciences Research Hub, 82 Wood Lane, London W12 0BZ, United Kingdom; Center for Biophysics and Quantitative Biology, University of Illinois at Urbana-Champaign, Urbana, Illinois 61801, USA; Department of Physics and Beckman Institute for Advanced Science and Technology, University of Illinois at Urbana-Champaign, Urbana, Illinois 61801, USA; Department of Chemistry, Imperial College London, Molecular Sciences Research Hub, 82 Wood Lane, London W12 0BZ, United Kingdom and Thomas Young Centre for Theory and Simulation of Materials, Imperial College London, South Kensington Campus, London SW7 2AZ, United Kingdom

**Keywords:** Biological Physics, Chemical Physics, Soft Matter

## Abstract

In solution, DNA, the “most important molecule of life,” is a highly charged macromolecule that bears a unit of negative charge on each phosphate of its sugar-phosphate backbone. Although partially compensated by counterions (cations of the solution) adsorbed at or condensed near it, DNA still produces a substantial electric field in its vicinity, which is screened by buffer electrolytes at longer distances from the DNA. This electric field is experienced by any charged or dipolar species approaching and interacting with the DNA. So far, such a field has been explored predominantly within the scope of a primitive model of the electrolytic solution, not considering more complicated structural effects of the water solvent. In this paper, we investigate the distribution of electric field around DNA using linear response nonlocal electrostatic theory, applied here for helix-specific charge distributions, and compare the predictions of such a theory with specially performed, fully atomistic, large-scale, molecular dynamics simulations. Both approaches are applied to unravel the role of the structure of water at close distances to and within the grooves of a DNA molecule in the formation of the electric field. As predicted by the theory and reported by the simulations, the main finding of this study is that oscillations in the electrostatic potential distribution are present around DNA, caused by the overscreening effect of structured water. Surprisingly, electrolyte ions at physiological concentrations do not strongly disrupt these oscillations and are rather distributed according to these oscillating patterns, indicating that water structural effects dominate the short-range electrostatics. We also show that (i) structured water adsorbed in the grooves of DNA leads to a positive electrostatic potential core relative to the bulk, (ii) the Debye length some 10 Å away from the DNA surface is reduced, effectively renormalized by the helical pitch of the DNA molecule, and (iii) Lorentzian contributions to the nonlocal dielectric function of water, effectively reducing the dielectric constant close to the DNA surface, enhance the overall electric field. The impressive agreement between the atomistic simulations and the developed theory substantiates the use of nonlocal electrostatics when considering solvent effects in molecular processes in biology.

## I. INTRODUCTION

**FIG. 1.**
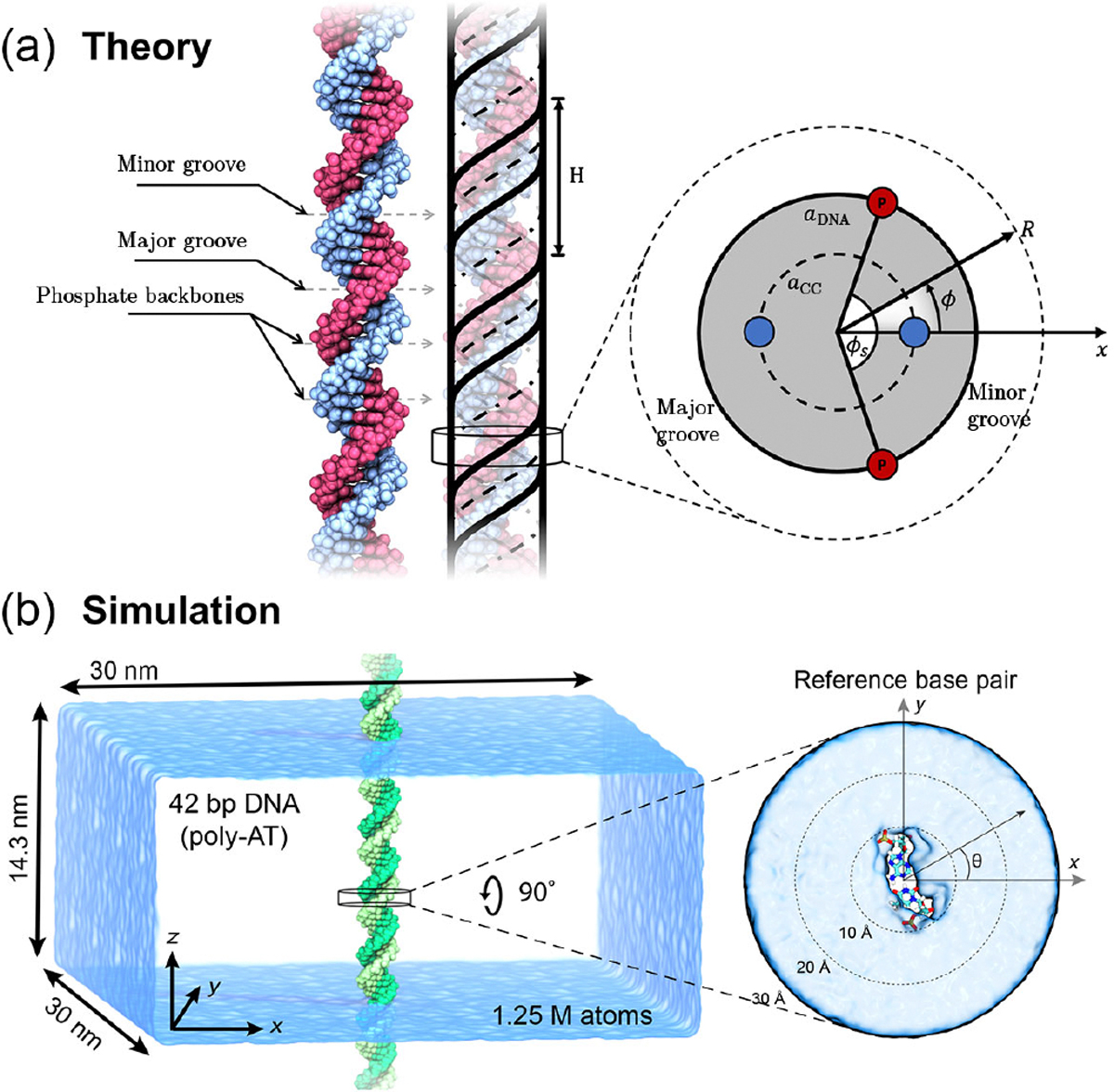
Comparison between theory and simulation models for a DNA molecule. (a) Illustration of DNA surface charge pattern, consisting of negatively charged helical lines of phosphates (solid lines) and positively charged counterions adsorbed in the major (dot-dashed lines) and minor (dashed lines) grooves. Such an ideal helical surface charge pattern closely corresponds to poly-AT DNA, which has helical pitch H≈34Å. On the right of diagram (a), we show a cross section of the DNA molecule at z=0, displaying the polar coordinate system (R,ϕ) we use in this work. Phosphates (red circles) sit at a radius of $a_{\text{DNA}}\approx 10\;Å$, where $\phi_{s}$ is the azimuthal width of the minor groove. Specifically adsorbed counterions (blue circles) sit within the DNA grooves at a radius of $a_{CC}\approx 5-7\;Å$. Not shown in the figure are the helical lines of structured water (the “water spine”), which also sit in the grooves at a radius of $a_{W}\approx 4-5\;Å$. (b) Illustration of the simulation system, consisting of a 42 base pair DNA (poly-AT) submerged in solvent (semitransparent surface). Each strand of the duplex is covalently linked to itself across the periodic boundary, making the DNA effectively infinite. A cylindrical volume of radius 3 nm, centered around the central base pair of the duplex, is taken as a reference. The inset defines a coordinate system perpendicular to the DNA’s helical axis.

A multitude of species interacting with DNA in solution experience its electric field. Indeed, this so-called “most important molecule” is an “electrostatic bomb.” Not actually an “acid” but usually a salt, DNA dissociates in aqueous solution, releasing its cations to the solution and retaining a unit of negative elementary charge on each phosphate of its sugar-phosphate backbone, resulting in two charges per 3.4 Å vertical rise (or per base pair) of the double helix. The double helices of these negative charges are screened by (i) the Debye cloud of buffer electrolyte, (ii) the DNA’s own, released counterions (which are in the minority in an electrolyte solution of physiological concentration around 0.154 M), and (iii) where applicable, by counterions (cations) of an added salt that specifically adsorb onto the DNA (see Fig. 1) or those that are condensed just in the narrow layer around DNA [1,2]. In such an environment, the “bomb” is neutralized, but the electric field, although exponentially decaying into the solution bulk, is still substantial in the vicinity of DNA within the range of a few nanometers. This field will act on any charged or polar species, and it thus plays an important role in DNA packing, interaction with proteins, and many other aspects of the vast genetic machinery.

Studies of the electric field of DNA have a long history, starting shortly after the discovery of DNA structure and function. The first popular model was the so-called poly-electrolyte model of DNA (for a review, see Refs. [1,2]). In this model, DNA was considered as a charged cylinder with characteristic DNA radius (approximately 1 nm) and mean surface charge density from the two phosphate strands ($\overset{‾}{\sigma }\approx 16.3\;\mu C\;{cm}^{-2}$), with the response of the surrounding ions to the presence of such a cylinder considered within approximations of various levels of complexity [3]. Such models helped to elucidate some basic effects in DNA biophysics [4] but were insufficient to unravel effects directly related to the helical structure and symmetry of DNA.

An attempt to understand the effects of the double-helical structure on the electric field of a DNA molecule was first made in 1978 [5], but systematic studies of such effects began in the late 1990s. This structure was initially studied in the context of DNA-DNA interactions [6], DNA in dense aggregates [7] and liquid crystals [8], DNA fibers (with reconsideration of structural information that can be extracted from the classical x-ray fiber diffraction patterns) [9], DNA supercoiling [10], and recognition of homologous genes [11] (for a detailed review, see Ref. [12]). These works have put weight on effects predominantly determined by the helical symmetry of DNA [13] (or violations and distortions of that symmetry [14]). Still, to a point, these works all rest on the implicit description of the solvent, describing its dielectric response by a macroscopic dielectric constant ε, as well as considering the ionic response in a simplified way based on the Debye-Bjerrum approximation [12]. A series of publications were devoted to accounting for the nonlinear response of the ionic subsystem, based on concepts of Wigner-crystal formation [15] and strong ionic correlations [3]; however, they did not consider the helicity of the DNA charge distribution, and again, neither went beyond a macroscopic description of the dielectric response of the surrounding water.

However, it has been known for several decades that macroscopic dielectric response is insufficient in the description of electrostatics in water. For example, let us consider a simple single ion: Submerging this ion in water will create a solvation sphere of bound solvent molecules, putatively resulting in an effective, reduced dielectric screening close to the ion. As polarization fluctuations in polar media are correlated in space (in the case of water, by its hydrogen bond network), this effect will persist over a certain characteristic length intrinsic to the solvent, and the effective dielectric constant will return to its bulk value only far away from the ion, at distances longer than this characteristic length. Given that this dielectric constant is effectively distance dependent, it is natural to refer to the language of nonlocal electrostatics, where water polarization correlations are conveniently described by the wave-number-dependent dielectric function ε(k); in the linear response approximation, such an approach provides a framework within which we can analyze the effect of the water structure and dielectric response to any charged object.

Simple models of the dielectric response of water interpolate smoothly between macroscopic (ε≈80) and high-frequency ($\varepsilon_{*}\approx 3-5$) dielectric constants, which leads to additional exponentially decaying contributions associated with the water structure in the electrostatic potential distribution near a charged or polar species embedded in water. As such, these structural contributions of water will appear in the potential distribution near an electrode, in hydration forces between charged or polarized surfaces, etc. Exponentially varying “structural” contributions to the forces between objects at the nanoscale have been measured in many biologically relevant systems from lipid membranes to DNA and proteins, as well as between surfaces relevant in electrochemistry.

However, water is more complex than this. Detailed analysis of its dielectric response shows so-called “overscreening” effects in addition to these exponential correlations. This effect means that, in the first molecular layer around a charged species, the amount of bound countercharge is larger than that on the species; that excess is overcompensated by an opposite-sign charge in the next layer, and it continues going until the macroscopic limit of screening is reached. The effect of overscreening manifests as a peak in the wave-number-dependent Fourier component of the response function, $\chi (k)=\varepsilon_{*}^{-1}-\varepsilon (k{)}^{-1}$, which implies that there exists a region where the dielectric function ε(k) can be negative, thus leading to oscillations in the electrostatic potential around the species or in hydration forces. Such oscillations have been observed by Israelachvili and Pashley in 1983 [16] in their force measurements with surface force apparatus (SFA) between atomically flat mica surfaces. Such oscillations have also been seen even in the earliest computer simulations [17–21], which begs the following question: Why were these oscillations not seen previously in force experiments between lipid membranes or even differently prepared mica surfaces? In their paper, Israelachvili and Pashley alluded to the roughness of the surfaces in question; structural and thermal fluctuations can disrupt the water structure, leading to oscillation dysphasia. Such reasoning is logical; recently, this conjecture was substantiated by a systematic theoretical analysis [22].

These oscillations are currently receiving significant attention and are being measured more and more frequently in a number of sophisticated frequency modulated atomic force microscopy (FM-AFM) experiments where it is possible to bypass the effect of roughness under the so-called “solvent-tip approximation” [23,24]. However, the exact consequence and relevance of such oscillating features on phenomena such as ion adsorption and double-layer structure is still under debate [25]. For the case of DNA, its large relative size compared to the solvent molecules in combination with its double-helical structure and thermal fluctuations would lead one to naively believe that all these oscillations would be smeared out. However, recent FM-AFM experiments measuring the hydration structure of DNA have shown that we indeed see signatures of oscillations in the force patterns [26]. Such an observation is consistent with the idea of a “DNA water spine”: a stabilizing chiral superstructure that has also been experimentally measured in x-ray diffraction experiments [27]. Hence, when considering the complex environment surrounding a single DNA molecule, we should generally resort to a more sophisticated model that can incorporate these oscillating features.

More recently, the all-atom molecular dynamics (MD) simulation method has become a ubiquitous tool for accurate characterization of biomolecular systems [28]. The application of this method to the study of DNA systems has been challenging because the high charge of the DNA molecules combined with physiological salt concentrations requires larger systems and long simulation times to fully equilibrate the ion atmosphere. Nevertheless, early work has shown that a fully atomistic MD model can reproduce DNA electrostatics inferred from continuum simulations and DNA supercoiling measurements [29]. Soon after, however, the standard parametrization of ion–DNA interactions was found to be inadequate to reproduce experimentally measured DNA–DNA forces [30]. The force field model was then refined by introducing surgical corrections to cation–DNA phosphate interactions, producing a molecular force field model capable of quantitative reproduction of the DNA array data [31,32] and competitive ion binding experiments [33,34]. The model was then used to *predict* the effect of DNA methylation on DNA–DNA forces [35], suggesting a physical mechanism for guiding DNA condensation into microcompartments according to the DNA sequence [36]. Nonetheless, simulation studies of DNA interactions [37,38] and DNA assemblies [39,40] continue to be of interest.

With these computational advances in mind, the following questions remain: How much will things change when we abandon the previously established macroscopic models of DNA electrostatics and try to account for the water structure around DNA? What effect does the water structure have on the distribution of ions around DNA, and what does this mean for the “fine structure” of the electric field created by a DNA molecule in solution? Is a theoretical approach to this problem unrealistic, and are these results accessible only through all-atom MD simulations?

This article is a first attempt to answer some of these questions. We will incorporate a dielectric function inspired by a field-theoretical account of the water structure into the dielectric response of the electrolyte to the helical charge distribution representing DNA. While the dielectric response near a charge or charged surface can be represented as a local electrostatic result with a distance-dependent dielectric constant, this effective dielectric function strictly cannot be reused between different charge configurations, as has been attempted in the past [41,42]. Thus, the nonlocal electrostatic approach, which in the linear response formalism is more general, is employed here [43]. Calculating the electrostatic potential, electric field, and charge density, we will analyze results both in electrolyte solution and in a hypothetical “pure water” case to understand the nature of the electrostatics of the system. After those tasks are achieved, we will perform fully atomistic molecular dynamics simulations of DNA, water, and electrolyte ions—an approach that, in principle, has its own extensive history [44,45]—and compare the results with those of the analytical theory.

We can conclude that the results of these two approaches appear in harmony with each other, which is especially important in the absence of direct experimental determination of the distribution of electric field around DNA. Specifically, the theoretical approach to the description of the dielectric response of the water solvent is based on (and limited by) linear nonlocal electrostatics [43,46]; thus, the importance of the verification of its predictions by fully atomistic computer simulations is obvious.

The structure of this article is as follows. Before presenting the analytical theory for the calculation of electric field about DNA, we first provide an “elevator pitch” of the principles of nonlocal electrostatics, illustrating and discussing its predictions for species much simpler than DNA. We then proceed to the basics of the analytical theory of the electric field of DNA in solution and present its results and predictions. Next, we describe the setup and parameters of the simulations performed in this study. Then, having covered the foundations of both the theoretical and computational methods, we present the results of the simulations and compare them to the predictions of the theory, discussing the consequences and findings of this in-depth study into the electrostatics of this most important molecule.

## II. NONLOCAL ELECTROSTATICS: A BIRD’S-EYE VIEW

In this work, we use the language of nonlocal electrostatics to understand the effect of structured water surrounding a charged object. To give the reader more context as to the applicability and validity of this generalization of classical electrostatics, we first provide a brief review of the main concepts of the approach below, applying it to one of the simplest models of an ion, the Born sphere. We use this method to show the degree of complexity required when more complicated features, such as oscillations in the water structure, are considered. Note that Gaussian units are used throughout the article in all mathematical formulas.

### A. Basic equations of nonlocal electrostatics

When we consider spatially correlated media, the displacement field D and polarization density field P at a point r are not simply proportional to the electric field E as in the constitutive relations of classical electrostatics, i.e., D(r)=εE(r) and P(r)=χE(r), where ε and χ are, respectively, the dielectric constant and dielectric susceptibility of the medium. Generally, D(r) and P(r) must depend on the electric field in the surrounding space of that point because there are spatial correlations in the system; in other words, the orientation of one dipole in an electric field in space depends on how its surrounding dipoles are oriented; i.e., it depends on the value of the electric field in the volume around the point r extending to the range of spatial correlation of polarization fluctuations. Hence, the central idea of nonlocal electrostatics is the generalization of these constitutive relations of classical electrostatics into the nonlocal form:

(1) $$D_{\alpha }(r)=\sum_{\beta }\int dr^{\prime }\varepsilon_{\alpha \beta }\left(r-r^{\prime }\right)E_{\alpha \beta }\left(r^{\prime }\right),$$

where the subscripts denote Cartesian components, α,β=x,y,z. The above-mentioned correlations manifest themselves in the kernel of the relation, $\varepsilon_{\alpha \beta }\left(r-r^{\prime }\right)$, the so-called non-local dielectric tensor. In the limit of macroscopic electrostatics, $\varepsilon_{\alpha \beta }\left(r-r^{\prime }\right)=\varepsilon \delta_{\alpha \beta }\delta \left(r-r^{\prime }\right)$, where $\delta_{\alpha \beta }$ is the Kronecker delta, and $\delta \left(r-r^{\prime }\right)$ is the Dirac delta function (such a form reduces this general nonlocal relation to the local expression). In homogeneous and isotropic media, it is convenient to instead consider the Fourier transform of this tensor, $\varepsilon_{\alpha \beta }(k)$, or, more precisely, its longitudinal component:

(2) $$\varepsilon_{\|}(k)=\sum_{\alpha \beta }\frac{k_{\alpha }k_{\beta }}{k^{2}}\varepsilon_{\alpha \beta }(k),$$

where k=|k|, which we will express as ε(k) below for brevity.

The potential of the field produced by an arbitrary charge distribution in a uniform medium is determined by Gauss’ law, $\nabla \cdot D=4\pi \rho_{\text{ext}}(r)$. After the substitution of Eq. (1) with E(r)=-∇φ(r), one has

(3) $$\sum_{\alpha \beta }\frac{\partial }{\partial r_{\alpha }}\int dr^{\prime }\varepsilon_{\alpha \beta }\left(r-r^{\prime }\right)\frac{\partial }{\partial r_{\beta }}\varphi \left(r^{\prime }\right)=-4\pi \rho_{ext}\left(r\right).$$

We can easily resolve this equation with respect to the potential φ. With the following definitions of the forward and inverse Fourier transforms of any function f(r),

(4) $$\tilde{f}(k)=\int drf(r)e^{-ik\cdot r},\;f(r)=\frac{1}{(2\pi {)}^{3}}\int dk\tilde{f}(k)e^{ik\cdot r}$$

as well as Eq. (2), we obtain an expression for the potential produced by a rigid distribution of external charges immersed in a nonlocal solvent,

(5) $$\tilde{\varphi }\left(k\right)=\frac{4\pi }{k^{2}\varepsilon \left(k\right)}{\tilde{\rho }}_{ext}\left(k\right).$$

From this expression, the first step we need is to determine the form of ε(k) for the solvent. In the past, the so called “Lorentzian” form of the dielectric function that interpolates between the limiting behavior at small and large k was widely used. Such an expression for ε(k) corresponds to purely exponentially decaying correlations in the system, allowing one to rationalize different experimental observations in a number of electrochemical systems [43,46]. However, this “Lorentzian model” fails to capture the full complexity of the system; it does not describe the overscreening mode in the dielectric response functions ubiquitous to polar liquids, where ε(k)<0. We can write a general expression for the dielectric function as follows:

(6) $$\varepsilon \left(k\right)=\frac{1}{\frac{1}{\varepsilon_{*}}-\left(\frac{1}{\varepsilon_{*}}-\frac{1}{\varepsilon }\right)\tilde{F}\left(k\right)},$$

where $\varepsilon_{*}\approx 3-5$ is the short-range dielectric constant. Within the Lorentzian model, $\tilde{F}(k)=1/\left(1+\Lambda^{2}k^{2}\right)$, where Λ is the correlation length of the polarization fluctuations in the liquid. This model does not consider finer effects in the k spectrum, leading to overscreening. Taking inspiration from a dielectric function derived from an extended phenomenological Landau-Ginzburg expansion in the polarization density of water (see Ref. [22] for details), we use the following expression, first proposed in Ref. [47]:

(7) $$\tilde{F}(k)=\frac{\gamma }{1\;+\;\Lambda_{1}^{2}k^{2}}\;\;+\frac{(1\;-\;\gamma ){\left(1\;+\;\Lambda_{2}^{2}Q^{2}\right)}^{2}}{\left(1\;+\;{\left(k\Lambda_{2}\;-\;Q\Lambda_{2}\right)}^{2}\right)\left(1\;+\;{\left(k\Lambda_{2}\;+\;Q\Lambda_{2}\right)}^{2}\right)}.$$

Such a form for the dielectric function accounts for a number of fine-structure effects in water. This case is particularly clear if we examine the poles of Eq. (7). There are six roots of the denominators, two of which are imaginary, located at $k=\pm i/\Lambda_{1}$, and the rest are complex, located at $k=\pm Q\pm i/\Lambda_{2}$; here, $Q,\;\Lambda_{1}$, and $\Lambda_{2}$ are related to the different correlation lengths in the system, and we take their values from fits to a simulated bulk water dielectric response function [22,48]. The two imaginary poles describe exponential (also known as Lorentzian) correlations, with a characteristic decay length $\Lambda_{1}\approx 3.67\;Å$. The complex poles describe decaying oscillating correlations, with a characteristic decay length $\Lambda_{2}\approx 1.77\;Å$ and oscillation period $\Lambda_{o}=2\pi /Q\approx 2.13\;Å$. The partitioning coefficient γ determines the relative strength of these contributions to the overall dielectric response. Here, we have chosen parameters such that ε(k) reproduces the response function for TIP3P water, the model used in the following DNA simulations (see Sec. V). It is important to note this choice when comparing the results of the theory against simulations performed with such rigid bond models that neglect internal degrees of freedom of the water molecule, such as electronic polarizability and bond vibrations. However, by accounting for partially charged hydrogen and oxygen atoms in the water molecule, an approximate value can be calculated for $\varepsilon_{*}$. For TIP3P water, it can be estimated that $\varepsilon_{*}\approx 5.75$. However, when more detailed models of the solvent are used in simulation, we must consider more carefully the value of this $\varepsilon_{*}$, as well as include the varying spatial dispersion of these internal degrees of freedom.

### B. Solvent response to the electric field of simple ions

Rather than diving headfirst into the more complicated case of DNA, we will begin by studying a single ion. The simplest model one can adopt for this purpose is the Born sphere (BS) model, which has been widely used in many electrostatic calculations in the past. Here, the charge of the ion is localized on a sphere of radius a, such that

(8) $$\rho_{BS}\left(r\right)=\frac{Ze}{4\pi a^{2}}\delta \left(\left|r\right|-a\right),$$

where Z is the valency of the ion, and e is the elementary charge. Such a model is quite crude; simple arguments from quantum mechanics tell us that a hard sphere of charge is a fairly poor model of an ion. First, given the presence of directional orbitals surrounding the ion, the charge distribution is generally not spherically symmetric. Second, electrons are not localized on an infinitely thin sphere but rather can be thought of as “smeared” along the radial direction. We therefore consider the smeared Born sphere (SBS) model introduced in Ref. [48]. This model is one of the simplest modifications of the Born model, neglecting anisotropy in the charge distribution. The charge distribution of such a sphere is given by

(9) $$\rho_{SBS}(r)=\frac{ZeN_{SBS}}{4\pi }e^{-||r|-a|/\eta }.$$

Here, a is the position of maximum charge density (effective ion radius), and η is the smearing parameter. We determine the normalization factor $N_{\text{SBS}}$ from the condition that $\int_{V}\rho (r)dr=q$, yielding

(10) $$N_{SBS}=\frac{1}{2\eta \left(a^{2}\;+\;\eta^{2}\left(2\;-\;e^{-a/\eta }\right)\right)}.$$

The classical Born model is a limiting case of this smeared model, as the smearing parameter η→0. The Fourier transforms of these charge distribution models are given by

(11) $${\tilde{\rho }}_{BS}\left(k\right)=Ze\frac{\sin\left(ka\right)}{ka},$$

(12) $${\tilde{\rho }}_{SBS}(k)=2ZeN_{SBS}\left\{\frac{\eta a\;sin(ka)}{k\left(1\;+\;\eta^{2}k^{2}\right)}+\frac{\eta^{3}(2cos(ka)-e^{\;-\;a/\eta })}{{\left(1\;+\;\eta^{2}k^{2}\right)}^{2}}\right\},$$

where we clearly see that Eq. (13) reduces to Eq. (12) in the case where η→0.

#### 1. Electrostatic potential

To calculate the electrostatic potential around a spherically symmetric ion, we can simply take the spherical 3D inverse Fourier transform of Eq. (5), yielding the nonlocal electrostatic formulation,

(13) $$\varphi \left(r\right)=\frac{2Ze}{\pi }\int_{0}^{\infty }\frac{dk}{\varepsilon \left(k\right)}\frac{\sin\left(kr\right)}{kr}{\tilde{\rho }}_{ext}\left(k\right).$$

From this formula, we can also define the screening function S(r)=εrφ(r)/Ze, which yields the deviation from Coulomb’s law for the electrostatic potential.

**FIG. 2.**
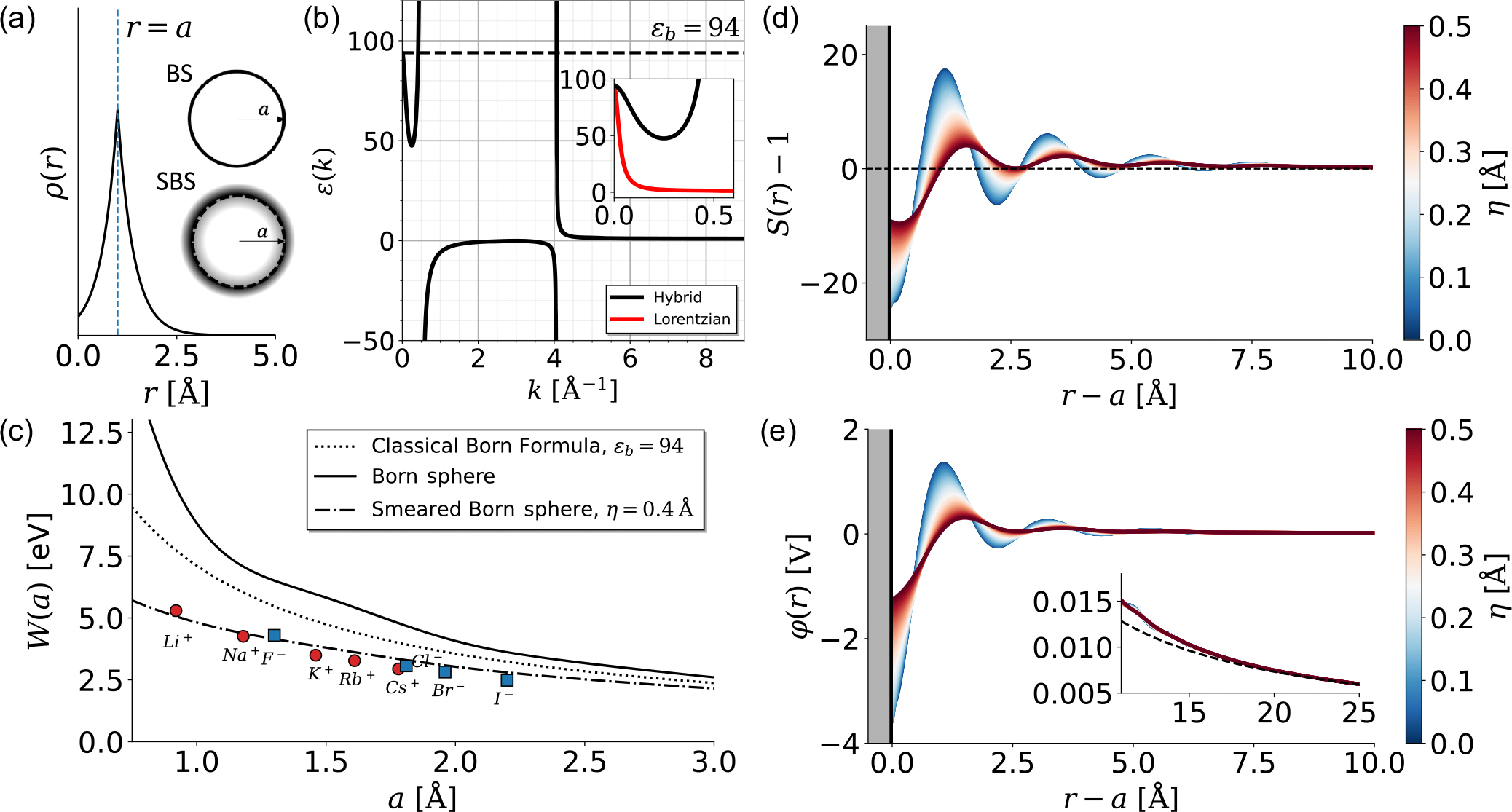
Nonlocal electrostatics results for a smeared Born sphere with a hybrid dielectric function parametrized to TIP3P and unit elementary charge, i.e., Z=1. (a) Model charge distributions for a=1Å and η=0.4Å. The vertical dotted blue line represents the BS model, and the solid black line represents the SBS model. (b) Hybrid model for the dielectric function of water, defined by Eqs. (6) and (7), parametrized for TIP3P water, with $\Lambda_{1}=3.67\;Å,\;\Lambda_{2}=1.77\;Å,\;\Lambda_{o}=2\pi /Q=2.13\;Å,\;\gamma =0.05$, and $\varepsilon_{*}=1$. At k=0,ε(k=0)=ε=94 for TIP3P water, as shown by the horizontal dotted line. The inset shows a comparison between the hybrid and Lorentzian models in the small-k region. Panels (c)–(e) all show quantities calculated using the model of ε(k). (c) Theoretical calculations against experimental measurements for hydration energy against ion radius. Experimental data for the hydration energy of the presented ions were taken from Ref. [50], with their Gourary-Adrian radii [51,52]. We clearly see that the classical Born formula and the hard BS model overestimate the hydration energy significantly. However, when smeared, the theoretical predictions match the experiment almost exactly. (d),(e) Screening function and electrostatic potential distribution, respectively, and how they vary with the smearing parameter η. The inset of panel (e) shows convergence with the classical Coulomb law at distances larger than around 20 Å, where the effect of polarization correlations disappears.

Limitations of this equation are as follows: (i) linear response of the dielectric medium to the electric charge and (ii) neglect of excluded volume of the charged object itself (the so-called “embedded charge approximation”). These limitations were both analyzed in Ref. [49], where it was shown that, very close (within one molecular diameter) to the ion, both effects lead to substantial differences in the distribution of electrostatic potential. Hence, we must acknowledge that the results we obtain through this non-local formalism within the range of one molecular diameter (here, about 2.5 Å for water) should be considered with caution. As can be seen in Figs. 2(d) and 2(e), these limitations manifest as a possible artefact, where we see inversion in the sign of the potential and screening factor very close to the ion surface.

#### 2. Free enthalpy of solvation

The free enthalpy of solvation, which we term here the hydration energy, is generally given as the difference between the electrostatic energy in a vacuum and in the solvent:

(14) $$W=\frac{1}{2}\int_{V}dr\left[\varphi_{0}\left(r\right)-\varphi \left(r\right)\right]\rho_{ext}\left(r\right).$$

Using Eq. (13) above for the electrostatic potential, we obtain [43]

(15) $$W(a)=\frac{1}{\pi }\int_{0}^{\infty }dk{\tilde{\rho }}_{ext}(k;a{)}^{2}\left[1-\frac{1}{\varepsilon \left(k\right)}\right].$$

It is important to note that such expressions for the hydration energy assume that the solvent penetrates inside the charge distribution (also known as the embedded charge distribution approximation [43,48]); such an assumption works reasonably well as an interpolation: When the ion radius is small and nonlocal effects are the largest, the amount of solvent “within the ion” is negligible, whereas for larger ions, the nonlocal effect diminishes and the assumption that the solvent sits inside the ion bears no importance, as in the classical Born formula. Note that, in the limit where ε(k)=ε=const and $\tilde{\rho }(k;a)\to {\tilde{\rho }}_{BS}(k)$, Eq. (15) reduces to the classical Born formula:

(16) $$W\left(a\right)=\frac{Z^{2}e^{2}}{2a}\left(1-\frac{1}{\varepsilon }\right).$$

Plotting the hydration energy against ion size in Fig. 2(c), we see that for large ions, the nonlocal expression for the Born sphere approaches the classical expression asymptotically. It is well known that the classical Born formula overestimates the hydration energy for ions [Fig. 2(c)] because of the approximation that ε(k)≡ε in all space, including in the vicinity of the ion, not accounting for nonlocal effects. When we calculate this hydration energy for a Born sphere in a nonlocal medium within the Lorentzian approximation, the value of the hydration energy successfully reduces to experimental values [43]. However, when we try to use more sophisticated models that account for overscreening, the overestimation worsens [Fig. 2(c)]. This result was shown to be an artefact of the BS model [48]. Calculating the hydration energy for the more realistic SBS model allows us to match the experimental results much more closely by simply smearing the charge slightly [48], where we have set η=0.4Å for all ions. Of course, each ion will have its own characteristic η, so it is not accurate to simply apply a constant value for all ionic radii. However, this example just shows the absolute importance of introducing smearing in the charge distribution when the dielectric function accounts for overscreening oscillations.

### C. Interpolation approximation for electrolytes

Recently, a phenomenological model for the dielectric function of pure water was proposed [22]; however, the approach to include electrolyte ions in that work was only valid for small concentrations so as to not violate the Dolgov-Kirzhnits-Maksimov (DKM) constraint [53] on the full electrolyte dielectric function, $\varepsilon_{c}(k)$. This constraint means that $\varepsilon_{c}(k)$ cannot enter the regime of $0<\varepsilon_{c}(k)\leq 1$ at any concentration of electrolytes. However, within the following approximation first used in Ref. [47], we can extend the dielectric function to account for both the solvent molecules and the ions present without worry about violating this DKM constraint.

Let us remind ourselves of the limiting behavior of the dielectric function; in the long-wavelength limit (small k), we recover macroscopic behavior, i.e., ε(k)→ε, whereas the short-wavelength limit (large k) probes the short-range correlations. For example, when we consider pure water, the wave numbers k∼2π/d, where d is the diameter of the water molecule, characterize the molecular packing effects in the solvent. This is the origin of the oscillation period $\Lambda_{o}(=d)$, obtained from the roots of ε(k). For much larger k,ε(k) will approach the short-range dielectric constant due to the electronic or infrared polarizability of the molecules, $\varepsilon_{*}$.

The analysis of the linear Poisson-Boltzmann equation for a binary monovalent solution yields the dielectric response function of ionic solutions in the long-wavelength limit (small k):

(17) $$\varepsilon_{c}\left(k\right)=\varepsilon \left[1+\frac{\kappa^{2}}{k^{2}}\right],$$

where $\kappa^{-1}=\lambda_{D}$ is the Debye screening length. The divergence at small wave numbers corresponds to the screening of the potential at distances larger than the Debye length. At smaller distances, the screening effect is negligible, and the dielectric response is only influenced by the water. This case will remain true even if we consider a more complicated expression for the water dielectric response, rather than ε. Hence, we can write a simple interpolated formula for the dielectric response by replacing ε with the full ε(k):

(18) $$\varepsilon_{c}\left(k\right)=\varepsilon \left(k\right)\left[1+\frac{\kappa^{2}}{k^{2}}\right],$$

where ε(k) is the pure water dielectric function that we can approximate by, e.g., Eqs. (6) and (7). Checking the limiting behavior of the expression, for long wavelengths, ε(k)→ε, and we recover Eq. (17). In the short-wavelength limit, the ionic contribution is neglected, and we recover the pure water response. By design, this interpolated formula is expected to work well if there is a separation of length scales: The Debye length is larger than the characteristic correlation lengths in the solvent and definitely much larger than the size of the solvent molecules. In this paper, we use this interpolated formula for the electrolyte dielectric function in the Poisson equation to solve for the potential around a DNA molecule.

Of course, writing a general dielectric function coupling the solvent to the ionic response is not an easy task. The approach outlined above, in the simplest way, is equivalent to replacing the inverse Debye length κ with some wave-number-dependent $\tilde{\kappa }(k)$, a sophistication that has received a lot of attention recently in attempt to combine the dielectric response of the solvent with the electrolyte ions [54–57]. However, in these works, the method by which we obtain $\tilde{\kappa }(k)$ is not straightforward, and it does not result in a simple formula. Hence, we use Eq. (18) here as a first approximation, keeping in mind its limitations.

In view of a number of the approximations used, such as the one inherent to Eq. (18), as well as the model for the dielectric function of water (although fitted to independent molecular dynamics simulations), we will systematically compare the predictions of the theoretical results with atomistic molecular dynamics simulations, which is the cornerstone of this paper.

## III. THEORY AND MODEL

### A. Basic equations

Let us now consider one infinitely long, cylindrical molecule with an arbitrary surface charge distribution. Here, we assume that an aqueous solution fills all the space, and the fixed charges are immersed in it. Given that water is able to penetrate within the grooves of DNA, this is not too crude an assumption to make. It should be noted, however, that this water has structure, resulting in a net polarization density, and therefore must be included in the model for the surface charge distribution as a “bound charge” contribution (see Sec. III B 3).

**FIG. 3.**
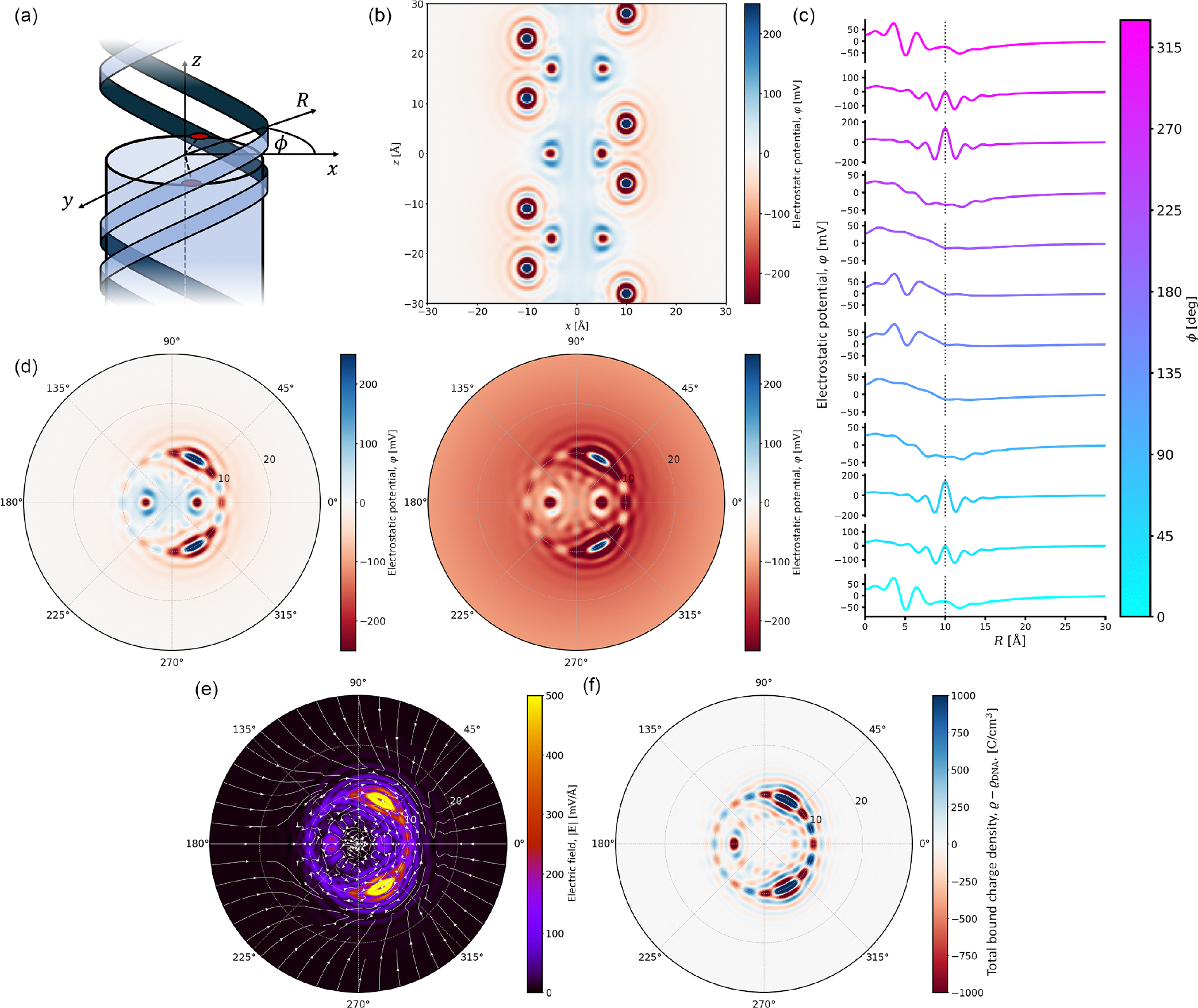
Theoretical results for the DNA electrostatic potential in solution. (a) Depiction of system and coordinate axes over which the results are plotted, also displaying the double-helical structure of the DNA molecule. For all plots, the parameters describing the bulk dielectric response of TIP3P are $\varepsilon =94,\;\varepsilon_{*}=5.75,\;\Lambda_{1}=3.67\;Å,\;\Lambda_{2}=1.77\;Å,\;\Lambda_{o}=2.13\;Å$, and γ=0.05. The parameters describing the DNA geometry and fluctuations are $h_{r}=3.4\;Å,\;\phi_{s}=2.2\;rad,\;a_{DNA}=10\;Å,\;\Delta_{eff}=0.25\;Å$, and $\delta a_{DNA}=0.5\;Å$. For the condensed counterion lines, $f_{1}=0.1,\;f_{2}=0.9,\;\delta z_{c1}=\delta z_{c2}=0.75\;Å,\;\Theta =0.1,\;a_{CC}=6\;Å$, and $\delta a_{CC}=0.75\;Å$. Bound charge distribution parameters are given by $P_{0}=-8\;\mu {Ccm}^{-2},\;w_{1}=1,\;w_{2}=0.75,\;a_{W}=5\;Å,\;\delta z_{w1}=0.75\;Å,\;\delta z_{w2}=0.75\;Å$, and $\delta a_{W}=0.75\;Å$. (b) Electrostatic potential distribution of a DNA molecule in the xz plane, for physiological concentrations ($c_{b}=0.154\;M,\;\kappa =0.118{\;Å}^{-1}$). Phosphate charges located at x=10Å induce oscillatory behavior in the electrostatic potential as a result of structured water, whereas condensed counterions and bound water in the grooves of DNA lead to a core of positive potential relative to the bulk. (c) Averaged line plots of the electrostatic potential distribution as we rotate around the DNA molecule. Each line is averaged over a 30-degree wedge ($\phi \pm 15^{\circ }$), starting at $\phi =15^{\circ }$. (d) Polar (R,ϕ) cross-sectional plots at z=0 of the electrostatic potential distribution for $c_{b}=0.154\;M$ and at $c_{b}=1\;mM$. (e),(f) Magnitude of electric field and bound charge density in pure water, i.e., at κ→0. Electric-field vector directions are drawn as white lines in diagram (e).

Using the approximation detailed above for the dielectric function of electrolytes, the Fourier transform of the electrostatic potential φ(r) created by any embedded charge distribution of volume charge density $\rho_{\text{ext}}(r)$ is given by

(19) $$\tilde{\varphi }\left(k\right)=\frac{4\pi }{\left(k^{2}\;+\;\kappa^{2}\right)\varepsilon \left(k\right)}{\tilde{\rho }}_{ext}\left(k\right),$$

where ${\tilde{\rho }}_{\text{ext}}(k)$ is the Fourier transform of $\rho_{\text{ext}}(r)$. As we are dealing with the surface charge density on a cylindrical surface, it is convenient for us to describe the external charge density in the molecular frame in a cylindrical coordinate system, (z,ϕ,r), associated with the molecular axis [see Fig. 3(a)]. There are multiple contributions to the external charge distribution (see the following subsections below)—to keep our formalism as general as possible, we can write the charge density of a given contribution ν as an arbitrary surface charge distribution, placed at a given radius $a_{\nu }$, such that

(20) $$\rho_{\nu }(z,\phi ,r)=\sigma_{\nu }(z,\phi )\delta \left(r-a_{\nu }\right).$$

It is then convenient for us to express it in the form

(21) $$\rho_{\nu }(r)=\frac{1}{(2\pi {)}^{2}}\sum_{n=-\infty }^{\infty }\int_{-\infty }^{\infty }dq\;e^{iqz}e^{in\phi_{R}}{\tilde{\rho }}_{\nu }(q,n,r)$$

so that

(22) $${\tilde{\rho }}_{\nu }(k)=a_{\nu }\sum_{n=-\infty }^{\infty }i^{n}{\tilde{\sigma }}_{\nu }(q,n)J_{n}\left(Ka_{\nu }\right)e^{-in\phi_{K}},$$

where ${\tilde{\sigma }}_{\nu }(q,n)$ is the Fourier transform of $\sigma_{\nu }(z,\phi )$, defined as

(23) $$\tilde{\sigma }(q,n)=\int_{0}^{2\pi }d\phi \int_{-\infty }^{\infty }dz\;e^{-iqz}e^{-in\phi }\sigma (z,\phi ),$$

and $J_{n}(x)$ is the nth-order Bessel function of the first kind. Plugging Eq. (22) into Eq. (19) and performing the inverse Fourier transform, we find the expression for the potential in real space:

(24) $$\varphi_{\nu }(r)=\frac{a_{\nu }}{\pi }\sum_{n=-\infty }^{\infty }\;\int_{-\infty }^{\infty }\;dq\int_{0}^{\infty }\;KdK\;\;\times e^{in\phi }e^{iqz}\frac{J_{n}(KR)J_{n}\left(Ka_{\nu }\right)}{\varepsilon \left(\sqrt{K^{2}\;+\;q^{2}}\right)\left(K^{2}\;+\;q^{2}\;+\;\kappa^{2}\right)}{\tilde{\sigma }}_{\nu }(q,n),$$

which will give us a result for a given ${\tilde{\sigma }}_{\nu }(q,n)$ and ε(k). In previous approaches to this problem, this expression has been extended to include the presence of low dielectric cylindrical cores, such that the charge distribution sits at the inner-core–water interface [58]. Considering that water is found in both the major and minor grooves and because we also consider spatial dispersion of the solvent, we will neglect such effects since accounting for them would have greatly complicated the theory. We will therefore continue to use the picture of the charge distribution immersed in a solution.

### B. Surface charge distribution model

The expression for the potential in Eq. (24) is valid for any charge distributed over concentric cylindrical surfaces of radii $a_{\nu }$. Previous formulations of the theory considered simple examples of infinitely thin, continuous, homogeneously charged helical lines (one or several), and a homogeneously smeared countercharge, both located at the cylinder-water interface [58], as well as smeared lines [6] or discrete charge arrays [13]. Given that we introduce a dielectric function that includes the overresponding behavior of water, these infinitesimally narrow lines of charge will introduce very large spatial oscillations of electrostatic potential into the system, as reasoned above when considering just single ions. We therefore need to take into account more complex effects associated with the finite size of the charged groups, on- and off-strand fluctuations around their regular positions on the helical strands, and inhomogeneous distributions of adsorbed countercharges, which will smear out these resonant overscreening effects and provide more realistic results.

#### 1. Backbone charge distribution

Here, we consider the DNA molecules to have ideal helical symmetry, which would imply that the relation

(25) $$\sigma (z,\phi )=\sigma \left(z+z^{\prime },\phi +gz^{\prime }\right)$$

must be satisfied for any $z^{\prime }$, where g=2π/H, and H is the helical pitch (for B-DNA, H=34Å). We see that there is an equivalency between z and ϕ: A change in z by $z^{\prime }$ is equivalent to rotating the molecule through an angle of $gz^{\prime }$. We therefore approximate the density of fixed surface charges, intrinsic to the helical molecules, by the following general expression, valid for any helical molecule:

(26) $$\sigma_{DNA}(z,\phi )=\frac{2\pi \overset{‾}{\sigma }}{N}\sum_{j=1}^{N}\;\int_{0}^{2\pi }\;d\phi^{\prime }\int_{-\infty }^{\infty }\;dz^{\prime }\psi \left(z-z^{\prime },\phi -\phi^{\prime }\right)\;\;\times \delta \left(\phi^{\prime }-\phi_{j}-g\left(z^{\prime }-z_{j}\right)\right),$$

where $\phi_{j}$ and $z_{j}$ describe the coordinates of the strands relative to the defined coordinate system, and N is the total number of helical strands (N=2 for B-DNA) on the molecule, where each strand is labeled by the index j. For DNA, if we define the coordinate system such that (z=0,ϕ=0) corresponds to the center of the minor groove, as in Fig. 1, $\left(z_{1},\phi_{1}\right)=\left(0,-\phi_{s}/2\right)$ and $\left(z_{2},\phi_{2}\right)=\left(0,\phi_{s}/2\right)$, where $\phi_{s}=0.8\pi$ is the width of the minor groove. We assume that the fixed charges are associated with surface groups centered on helical strands. We account for the finite size of charged groups by introducing the form factor ψ(z,ϕ), normalized as ∬ψ(z,ϕ)dϕdz=1, such that ψ(q=0,n=0)=1, where $\tilde{\psi }(q,n)$ is the cylindrical Fourier transform of ψ(z,ϕ). Taking the Fourier transform of this expression, we find that, for B-DNA,

(27) $${\tilde{\sigma }}_{DNA}(q,n)=4\pi^{2}\overset{‾}{\sigma }\tilde{\psi }(q,n)\delta (q+ng)cos\left(\frac{n\phi_{s}}{2}\right).$$

In the case of thin line charges, ψ(z,ϕ)=δ(z)δ(ϕ), and hence $\tilde{\psi }(q,n)=1$. For the simplest fluctuation case of Gaussian disorder in both ϕ and z, we can write

(28) $$\psi (z,\phi )=\frac{1}{2\pi \delta z\delta \phi erf\left[\frac{\pi }{\sqrt{2}\delta \phi }\right]}exp\left[-\frac{z^{2}}{2\delta z^{2}}\right]exp\left[-\frac{\phi^{2}}{2\delta \phi^{2}}\right],$$

where δz and δϕ are the effective half-width of the distributions, related to the atomic form factors of the charged groups and the mean-square amplitude of their fluctuations around the “helical lines.” Taking the Fourier transform, in the limit where δϕ≪π, we obtain

(29) $$\tilde{\psi }(q,n)=exp\left[-\frac{1}{2}q^{2}\delta z^{2}\right]exp\left[-\frac{1}{2}n^{2}\delta \phi^{2}\right].$$

Considering that in Eq. (27) this expression enters in a product with the Dirac delta function, δ(q+ng), for $\tilde{\psi }(q,n)$ we can use a simpler expression,

(30) $$\tilde{\psi }\left(q,n\right)=\exp\left[-\frac{1}{2}n^{2}g^{2}\Delta_{eff}^{2}\right],$$

where

(31) $$\Delta_{eff}=\sqrt{\delta z^{2}+\frac{\delta \phi^{2}}{g^{2}}}$$

is an “effective” half-width of the distribution. Note that Gaussian on- or off-strand fluctuations of the groups around their regular positions on the strands, static or dynamic, result in similar form factors. The incorporation of these form factors into the theory may further cover the effects due to the finite size of the groups, quenched Gaussian disorder, and/or Debye-Waller factors. Below, in Sec. III D, we will show how similar smearing effects in the radial direction may be accounted for. Here, however, we start first with the case of “on-the-surface” smeared form factors, described by Eq. (30).

#### 2. Condensed counterion distributions

Highly charged helical molecules, such as DNA, cause adsorption (condensation) of counterions onto their surfaces. The adsorbed counterions are typically more mobile than the fixed surface charges described above, and they may either surround the fixed charges or bind into grooves between the strands formed by fixed charges. We therefore approximate the surface density of adsorbed charges by the inhomogeneously smeared charge density $\sigma_{c}(z,\phi )$, which follows the same basic helical symmetry as the charged strands. The most general expression we can write for the charge density that satisfies this symmetry is

(32) $$\sigma_{c}\left(z,\phi \right)=2\pi {\overset{‾}{\sigma }}_{c}\int_{-\infty }^{\infty }dz^{\prime }p\left(z-z^{\prime }\right)\delta \left(\phi -gz^{\prime }\right),$$

where we have defined the coordinate system in the same way as above, with (z=0,ϕ=0) corresponding to the center of the minor groove. Here, the subscript c labels parameters for counterions, and ${\overset{‾}{\sigma }}_{c}$ is their average surface charge density. We can relate this case to the average surface charge density of the DNA $\overset{‾}{\sigma }$ through ${\overset{‾}{\sigma }}_{c}=-\Theta \overset{‾}{\sigma }$, where Θ is the degree of the overall charge compensation by condensed counterions. Note that p(z) is the probability density of counterion adsorption at the axial distance z from the center of the minor groove, normalized such that its Fourier transform at q=0 is $\tilde{p}(q=0)=\int p(z)dz=1$. For example, setting p(z)=δ(z) corresponds to the counterions sitting exactly in the middle of the minor groove. Taking the Fourier transform of Eq. (31), we find

(33) $${\overset{‾}{\sigma }}_{c}\left(q,n\right)=4\pi^{2}{\overset{‾}{\sigma }}_{c}\delta \left(q+ng\right)\tilde{p}\left(q\right).$$

Here, we can analyze a specific four-state counterion adsorption pattern, where the smeared probability density is written as

(34) $$p(z)=\underset{\text{in}\;\text{the}\;\text{minor}\;\text{groove}}{\underbrace{\frac{f_{1}}{\sqrt{2\pi }\delta z_{c1}}e^{-\frac{z^{2}}{2{\left(\delta z_{c1}\right)}^{2}}}}}+\underset{\text{in}\;\text{the}\;\text{major}\;\text{groove}}{\underbrace{\frac{f_{2}}{\sqrt{2\pi }\delta z_{c2}}e^{-\frac{(z-H/2{)}^{2}}{2{\left(\delta z_{c2}\right)}^{2}}}}}\;\;+\underset{\text{on}\;\text{the}\;\text{strands}}{\underbrace{\frac{f_{3}}{2\sqrt{2\pi }\delta z_{c3}}\left\{e^{-\frac{{\left(z-H\phi_{s}/4\pi \right)}^{2}}{2{\left(\delta z_{c3}\right)}^{2}}}+e^{-\frac{{\left(z+H\phi_{s}/4\pi \right)}^{2}}{2{\left(\delta z_{c3}\right)}^{2}}}\right\}}}+\underset{\text{smeared}}{\underbrace{\frac{2\pi }{L}f_{4}}},$$

where different $f_{i}$ denote fractions of counterions adsorbed in different preferential locations, such that $\sum_{i}f_{i}=1$. As labeled in Eq. (33), these locations are as follows: near the center of the minor groove $\left(f_{1}\right)$, near the center of the major groove ($f_{2}$), near the charged strands ($f_{3}$), and randomly distributed along the cylinder surface ($f_{4}$). Here, we assume that the distribution of ions around each preferential adsorption location is Gaussian, with half-width $\delta z_{i}$. Despite writing the expression for all possible sites in Eq. (34), for simplicity, in the model below, we will only consider ions condensed in the minor and major grooves. Calculating the necessary Fourier transforms, we find that

(35) $${\tilde{\sigma }}^{c}(q,n)=4\pi^{2}{\overset{‾}{\sigma }}_{c}\delta (q+ng)\;\;\times \left(f_{1}e^{-\frac{1}{2}n^{2}g^{2}\delta z_{c1}^{2}}+f_{2}(-1{)}^{n}e^{-\frac{1}{2}n^{2}g^{2}\delta z_{c2}^{2}}\right).$$

#### 3. Bound charge distribution

Having described the response of water molecules only through the solvent’s nonlocal dielectric function ε(k), we do not take into account water molecules specifically adsorbed (bound) to DNA, particularly in the grooves. As mentioned in the Introduction, there exists a chiral “spine of hydration” that sits in the minor groove. The water molecules that contribute to this spine form a helical superstructure, with their hydrogens pointing towards the central axis. This result leads to a line of nonzero polarization density that we must take into account through an additional contribution to $\tilde{\sigma }(q,n)$.

Indeed, in terms of the volume charge densities, the total charge density is $\rho =\rho_{f}+\rho_{b}$, where $\rho_{f}(r)=\sigma_{\text{DNA}}(z,\phi )\times \delta \left(r-a_{DNA}\right)+\sigma^{C}(z,\phi )\delta \left(r-a_{CC}\right)$ is the free charge density associated with the fixed DNA and counterion charges, and $\rho_{b}$ is the bound charge associated with specifically adsorbed dipoles. However, basic electrostatic identities reveal a different relationship between the volume and surface bound charge densities,

(36) $$\rho_{b}\left(r\right)=-\sigma_{b}\left(z,\phi \right)\delta \left(r-a_{w}\right),$$

where $a_{w}$ is the radius of the cylinder along which the bound surface charge distribution sits. We can easily relate this case to the polarization density distribution, given that $\sigma_{b}=P\cdot \overset{ˆ}{n}$ [59]. For a cylindrical surface, we see that $\sigma_{b}(z,\phi )\equiv P_{⊥}(z,\phi )$, where $P_{⊥}(z,\phi )$ is the radial (normal) component of the polarization density. Hence, as above, we can write the bound charge distribution of the water spine in the grooves as

(37) $${\tilde{\sigma }}_{b}(q,n)=4\pi^{2}{\overset{‾}{P}}_{0}\delta (q+ng)\;\;\times \left(w_{1}e^{-\frac{1}{2}n^{2}g^{2}\delta z_{w1}^{2}}+(-1{)}^{n}w_{2}e^{-\frac{1}{2}n^{2}g^{2}\delta z_{w2}^{2}}\right),$$

where $w_{1}$ and $w_{2}$ are the relative fractions of the mean radial polarization density ${\overset{‾}{P}}_{0}$ associated with water adsorbed in the minor and major grooves, respectively, and $\delta z_{w1}$ and $\delta z_{w2}$ are the corresponding widths of their distributions about the center lines of the grooves.

Considering this contribution, we also account for the dielectric response of the first layer of water in the grooves of the DNA molecule, which elsewhere is considered in the linear response approximation.

### C. Electrostatic potential distribution due to DNA

Substituting Eqs. (27), (35), and (37) into Eq. (24), we obtain an expression for the electrostatic potential distribution in cylindrical coordinates. Writing the potential as a sum of contributions from the phosphates (DNA), the specifically adsorbed (condensed) counterions (CC), and the structured water (W), the full expression reads

(38) $$\varphi_{tot}=\varphi_{DNA}+\varphi_{CC}+\varphi_{W},$$

(39) $$\varphi_{DNA}=8\pi a_{DNA}\overset{‾}{\sigma }\sum_{n=0}^{\infty }\;\frac{e^{-\frac{1}{2}n^{2}g^{2}\Delta_{eff}^{2}}}{\delta_{n,0}\;+\;1}\cos\left[n\left(\phi -gz\right)\right]\cos\left[\frac{n\phi_{s}}{2}\right]{𝒲}_{n}\left(R;a_{DNA},\kappa \right),$$

(40) $$\varphi_{CC}=-8\pi a_{CC}\overset{‾}{\sigma }\Theta \sum_{n=0}^{\infty }\;\frac{f_{1}e^{-\frac{1}{2}n^{2}g^{2}\delta z_{c1}^{2}}\;+\;f_{2}(-1{)}^{n}e^{-\frac{1}{2}n^{2}g^{2}\delta z_{c2}^{2}}}{\delta_{n,0}\;+\;1}\cos\left[n\left(\phi -gz\right)\right]{𝒲}_{n}\left(R;a_{CC},\kappa \right),$$

(41) $$\varphi_{W}\;=-8\pi a_{W}{\overset{‾}{P}}_{0}\sum_{n=0}^{\infty }\;\frac{w_{1}e^{-\frac{1}{2}n^{2}g^{2}\delta z_{w1}^{2}}\;+\;w_{2}(-1{)}^{n}e^{-\frac{1}{2}n^{2}g^{2}\delta z_{w2}^{2}}}{\delta_{n,0}\;+\;1}\cos\left[n\left(\phi -gz\right)\right]{𝒲}_{n}\left(R;a_{W},\kappa \right).$$

As mentioned, for simplicity, we have only considered counterions condensed in the minor ($f_{1}$) and major ($f_{2}$) grooves in Eq. (40). It is clear here that the n=0 term corresponds to the potential around a homogeneously charged cylinder; thus, the n≥1 terms are usually referred to as the “helical harmonics” [12]. The general formula above can be applied for any case of specific counterion condensation on the DNA molecule. The electrostatic propagator ${𝒲}_{n}\left(R;a_{i},\kappa \right)$ describes how the potential varies in the radial direction, and for ε(k) given by Eqs. (6) and (7), it is calculated as

(42) $${𝒲}_{n}(R;a,\kappa )={\tilde{g}}_{\kappa }K_{n}\left({\tilde{\kappa }}_{n}R\right)I_{n}\left({\tilde{\kappa }}_{n}a\right)+\gamma {\tilde{g}}_{L}K_{n}\left({\tilde{q}}_{d1}^{n}R\right)I_{n}\left({\tilde{q}}_{d1}^{n}a\right)\;\;+(1-\gamma )Re\left\{{\tilde{g}}_{o}H_{n}^{\left(2\right)}\left[\left({\tilde{g}}_{n}^{R}-i{\tilde{g}}_{n}^{I}\right)R\right]\right.\;\;\left.\times J_{n}\left[\left({\tilde{g}}_{n}^{R}-i{\tilde{g}}_{n}^{I}\right)a\right]\right\}$$

for R>a, where $I_{n}(x)$ and $K_{n}(x)$ are the nth order modified Bessel functions of the first and second kind, respectively, and $H_{n}^{\left(2\right)}(x)$ is the nth order Hankel function of the second kind. In the case of R<a, we simply swap the positions of R and a in the Bessel functions. The definitions of all the parameters are given in Appendix A. In general, these parameters all depend on the roots of the denominator in the integrand of this integral. In the complex plane of K, one root is found at $K=\pm i\sqrt{\kappa^{2}+n^{2}g^{2}}$, and the rest are roots of $\varepsilon (\sqrt{K^{2}+n^{2}g^{2}})$; in the case of the approximation we use in Eq. (7), the latter are functions of the parameters $\Lambda_{1},\;\Lambda_{2}$, and Q, which relate to the characteristic correlation lengths in the system. For this approximation, the result of the integral is split into three terms, each involving certain characteristic lengths. By simply inspecting which lengths are involved with each term, we can gain a deeper understanding of the different contributions to the electrostatics in the system.

The first term arises from ion correlations in the system. Given the simple, linear, Debye-Hückel approximation used in this derivation, we see it follows a simple quasiexponential decay law where the Debye length $\kappa^{-1}$ is coupled with the DNA helical pitch. The second term arises from exponential water correlations (Lorentzian behavior) in the system, which is clear given the presence of $\Lambda_{1}$ in the decay length, also coupled with the DNA helical pitch. Finally, the oscillatory contributions are given by the third term. Given the complex arguments of the Hankel and Bessel functions, it is not possible to express these contributions in terms of commonly known special functions. However, we see that these contributions come from the complex root of ε(k), so the oscillation and decay lengths are related to $\Lambda_{2}$ and Q, again coupled with the DNA helical pitch. This finding is clear given the presence of the parameters ${\tilde{g}}_{n}^{R}$ and ${\tilde{g}}_{n}^{I}$ in the arguments of the Hankel and Bessel functions, and the fact that for $n\to 0,{\tilde{g}}_{n}^{R}\to Q$ and ${\tilde{g}}_{n}^{I}\to 1/\Lambda_{2}$ (see Appendix A for definitions).

In addition to the electrostatic potential, it is convenient for us to also calculate other electrostatic quantities, such as the electric field E and the total charge density in the system, ϱ. Having obtained the result above for the total electrostatic potential, it is trivial to calculate these quantities as $E=-\vec{\nabla }\varphi$ and Δφ=-4πϱ. Importantly, these quantities do not diverge in pure, electrolyte-free water, i.e., κ→0, which is not the case for the electrostatic potential; if we consider the simpler case of a homogeneously charged cylinder in a bulk dielectric, the electrostatic potential distribution diverges logarithmically at long distances from its axis. Hence, to study the electrostatics of a more pedagogical pure water case, we must consider the electric field and charge density. Of course, the electric field is also of interest per se, as it, to a high degree, determines the force with which DNA will interact with any charged objects.

### D. Radial smearing

In addition to the fluctuations of surface charge distributions in the z and ϕ directions, we now consider fluctuations in the radial direction. Looking again at our definition for the charge distribution in Eq. (20), one of the main assumptions is that the lines of charge sit on a cylinder at a fixed radius a. Such simplification would be suitable had we not included the overscreening effect in the solvent. However, we saw in our brief study of the Born sphere above that accounting for oscillations can lead to overemphasized resonance effects; it is also important to consider similar smearing of the DNA charge distribution in the radial direction, as it should further suppress the resulting amplitudes of oscillations in the electrostatic potential.

Allowing for smearing of the distribution in the radial direction, we can generalize the expression for the volume charge density of a given helical line ν as

(43) $$\rho_{\nu }\left(z,\phi ,r\right)=s_{\nu }\sigma_{\nu }\left(z,\phi \right)\zeta \left(r-{\overset{‾}{a}}_{\nu },\delta a_{\nu }\right),$$

where $s_{\nu }=\pm 1$ indicates the sign of the contribution; for free charge density (phosphates and adsorbed ions), $s_{\nu }=1$, and for bound charge density (adsorbed water), $s_{\nu }=-1$. As above, we can consider the case of Gaussian fluctuations, in which case, ζ takes the form of a truncated Gaussian distribution, as r>0:

(44) $$\zeta \left(r-{\overset{‾}{a}}_{\nu },\delta a_{\nu }\right)=\frac{\sqrt{2}\Theta (r)}{\sqrt{\pi }\delta a_{\nu }\left(1+erf\left[\frac{{\overset{‾}{a}}_{\nu }}{\sqrt{2}\delta a_{\nu }}\right]\right)}e^{-\frac{{\left(r-{\overset{‾}{a}}_{\nu }\right)}^{2}}{2\delta a_{\nu }^{2}}},$$

where ${\overset{‾}{a}}_{\nu }$ is the mean radius of the surface along which the helical line runs, $\delta a_{\nu }$ is the half-width of the distribution, and Θ(x) is the Heaviside step function. Following the derivation presented above for the electrostatic potential due to charge distributions on a cylindrical surface, we can write an extension of this result for the case of a radially smeared charge distribution,

(45) $$\overset{‾}{\varphi }\left(r\right)=\sum_{\nu }{\overset{ˆ}{\zeta }}_{\nu }s_{\nu }\varphi_{\nu }=\sum_{\nu }\int_{0}^{\infty }da_{\nu }s_{\nu }\varphi_{\nu }\left(r;a_{\nu }\right)\zeta \left(a_{\nu }-{\overset{‾}{a}}_{\nu },\delta a_{\nu }\right),$$

where we term $\overset{ˆ}{\zeta }$ the “smearing” operator, summation runs over all helical charge motifs ν associated with DNA (phosphate charges, adsorbed counterion charges, and adsorbed water bound charges), and $\overset{‾}{\varphi }$ is the smeared potential. By tuning the half-width of the distributions we smeared over, we can more precisely account for effects of thermal and structural fluctuations of the DNA molecule.

## IV. THEORETICAL RESULTS AND PREDICTIONS

Plotting Eqs. (38)–(41) and applying radial smearing as in Eq. (45), we obtain maps of the electrostatic potential distribution around the DNA molecule in Fig. 3. We plot the potential in three ways to showcase different behaviors and observations; see Fig. 3(a) to see how they relate to the structure of the double-helical molecule. First, in Fig. 3(b), we plot a slice in the xz plane through the center of the molecule. For the estimated parameters, we clearly see oscillations in the potential propagating from the double-helical phosphate lines running about the molecule. As discussed when considering the much simpler Born sphere case, the absolute value of the potential within one molecular diameter of the field source must not be taken literally, as we treat the DNA charge distribution within the embedded charge approximation and do not consider the nonlinear response of the medium to the excluded volume of the phosphates. Such approximations in combination with the overscreening dielectric response of the water therefore lead to the positive and negative “hotspots” on the phosphates and inside the groove, respectively.

However, while the presence of these oscillations is not a feature to ignore (it will be discussed in further detail below), what is particularly interesting here is the presence of a core of positive potential inside the DNA. Such an observation has previously been made in simulation studies of DNA as early as 1989 [60], but its physical origin is still under debate. Such an effect is also seen at the lipid bilayer-water interface. Some arguments seem to indicate that any positive potential inside nonaqueous systems (like inside the DNA duplex or the lipid bilayer) arises from quadrupolar contributions from water situated at the interface [61]. The quadrupolar moment is not explicitly considered here in the theory, and it is instead only partially accounted for by fitting to the simulated dielectric response functions of pure water. Thus, we do not expect quantitative accuracy of the theory inside the DNA. However, we qualitatively show here that such a positive potential can arise by accounting for adsorbed water by imposing helical lines of polarization density within the grooves of the DNA molecule.

When we consider the interactions of DNA in biology, they often appear with macromolecules far larger than the size of these oscillations, so they will often experience a more averaged electric field. The question then becomes whether these interacting molecules will even feel these oscillations. In Fig. 3(c), we plot the potential distribution averaged over 30-degree slices ($\phi \pm 15^{\circ }$) as we circle the molecule. Even over such a large slice, these oscillations are pronounced close to the minor groove and the phosphate strands. However, these oscillations appear to be weaker in magnitude in the vicinity of the major groove. Such reduced potential in this region compared to the minor groove side can perhaps lead to overall lower electrostatic repulsion, potentially providing a stronger impetus for proteins to approach and specifically bind in this region. Indeed, most DNA-binding proteins will bind to the major groove, where they have better access to the individual nucleotide bases to be “read” by the protein [62].

Figure 3(d) showcases the electrostatic potential as a polar (R,ϕ) map at both physiological concentration (0.154 M) and low concentration (1 mM). One clear difference when comparing the two is the absence of a positive core at low concentration, which indicates that this effect is not solely reliant on the behavior of water at the interface. Rather, it is a coupled ion-water effect, where the positive potential can arise from ions screening the phosphate charge from inside the double helix.

We also see that the oscillatory features remain unchanged between the low and physiological concentration regimes. While such an observation may be clear from examining the form of Eq. (42), this result is not a trivial one, and the significance of these oscillations is still under debate. A recent work on this electrostatic double-layer problem with a field-theoretical approach indicates that for millimolar concentrations, these oscillations trap ions, resulting in ion layering at the interface [22]. While the high concentration limit was not studied there, it is clear that ion correlations will take charge, affecting the oscillatory behavior in the electrostatic potential profile. Although these limits have been well studied in varying degrees of complexity, the behavior of the “intermediate” concentration regime is still unclear. Do physiological concentrations sit within this intermediate regime, and if so, how do the water-water and ion-ion correlation effects interfere or couple with each other? Our results seem to suggest that even at physiological concentrations, the electrostatics are still dominated by the solvent response to the DNA.

To strengthen this argument, we must make a comparison between physiological and pure water cases to directly observe the effect of electrolyte concentration. However, as we have already noted above, in pure water, i.e., as κ→0, it is clear that the potential diverges for the n=0 harmonic (i.e., for a homogeneous cylinder). Hence, here we only consider the electric field and charge density, expressions for which are given in Appendix B, derived from Eqs. (38)–(42). Plotting these electrostatic quantities in Figs. 3(e) and 3(f) for pure water, we see that these spatial oscillations are purely a consequence of the overscreening dielectric response of water. Note that in calculating the charge density, the contribution from the DNA surface charge distribution was subtracted, leaving behind only the bound charge density oscillations associated with water.

**FIG. 4.**
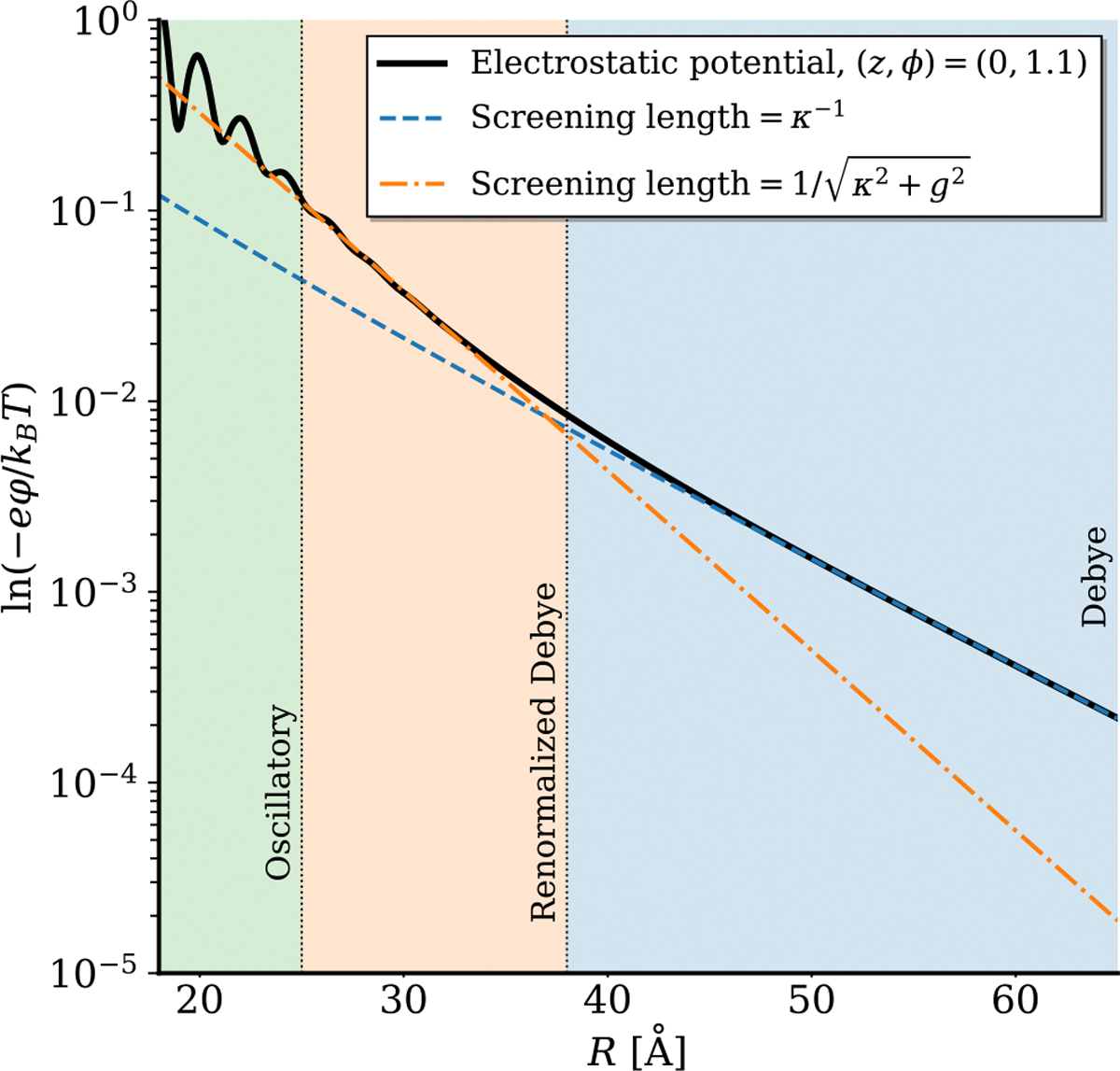
Analysis of the long-range Debye-like tail of the radial electrostatic potential distribution of a DNA molecule. The black curve is plotted for the same parameters as in Fig. 3, at physiological concentrations along (z,ϕ)=(0,1.1rad). The blue (dashed) and orange (dot-dashed) lines are exponential fits, $f(R)=Ae^{-R/\lambda }/\sqrt{R}$ with decay (screening) lengths $\lambda =\kappa^{-1}$ and $\lambda =1/\sqrt{\kappa^{2}+g^{2}}$, respectively. Such fits allow us to identify three screening regions of the DNA molecule: (i) the interfacial “oscillatory” regime where water dominates, (ii) the renormalized Debye-like regime, where the periodic helicity of the DNA molecule couples with the Debye length, overall reducing the screening length, and (iii) the Debye-like regime, where screening can be approximated by the Debye length of the electrolyte.

Finally, it is of interest to understand the screening of the electrostatic potential, as it determines the electrostatic forces experienced by other charged molecules further away from the DNA. By examining the Debye-like tail of the potential distribution, we can identify three regions as we move away from the DNA molecule: (i) the oscillatory “interfacial” region which is predominantly controlled by correlations in water, (ii) the steeper renormalized Debye tail with screening length $1/\sqrt{\kappa^{2}+g^{2}}$ (cf. Ref. [58]) which couples the helical pitch of the DNA to the Debye screening length, and (iii) the classical Debye tail with screening length $\kappa^{-1}$ (see Fig. 4). This finding is evident by fitting exponential curves to these different regions and comparing their gradients against the calculated potential curve. Indeed, such an exponential form would be appropriate to fit the potential, as the Bessel functions of the second kind in Eq. (41) are exponentially decaying in their asymptotic, large argument limit, such that ${𝒦}_{n}(x)∼e^{-x}/\sqrt{x}$. Overscreening is not the only important contribution in region (i). The overall field near the DNA is also enhanced by the Lorentzian contribution to the dielectric response. The slope around which oscillations take place in this region is determined by ${\tilde{q}}_{d1}^{n}$, which is greater than ${\tilde{\kappa }}_{n}$ (see Eq. (42), and Eqs. (A1) and (A2) in Appendix A).

We summarize the main findings of the theory developed in the first part of this paper below:

- At long distances (R>40Å), see the slowest decaying tail of electrostatic potential related to the uncompensated DNA charge. There, we approach the macroscopic limit, and the screening length is simply equivalent to the Debye length, $\kappa^{-1}$.
- At intermediate distances (25Å<R<40Å), we see the effect of the double-helical structure of DNA coupled to the Debye length, leading to a renormalized screening length of $1/\sqrt{\kappa^{2}+g^{2}}$, which is in line with the previous theoretical results based on the primitive model of electrolytes [58].
- At short distances (R<25Å), Lorentzian contributions to the dielectric response of water and, consequently, a reduced effective dielectric constant close to the DNA surface lead to a dramatic enhancement of the overall electric field.
- In this “short-distance” regime, we see strong spatial oscillations in the electrostatic potential. These oscillations are a consequence of the overscreening dielectric response of water to the phosphate charges. The amplitude of these oscillations is reduced when the helical lines of phosphate and adsorbed counterion charges are more smeared.
- Specifically adsorbed water molecules and counter-ions in the grooves of DNA lead to a positive core of electrostatic potential relative to the bulk.

Of course, these results strongly depend on the model of dielectric response of the electrolyte medium used, so we must either prove or disprove this linear response analysis by comparison with all-atom simulations, the details of which are described in the next section.

## V. SIMULATION SYSTEMS AND METHODS

### Summary of MD simulations—

We conducted MD simulations of a 42-bp DNA molecule immersed in an electrolyte solution, varying the composition of the latter. A typical system [Fig. 1(b)] consisted of about 1.2-million atoms and measured 30 nm × 30 nm × 14.3 nm. The DNA molecule was placed with its helical axis aligned along the z axis and was made effectively infinite by extending the covalent bonds of the DNA backbone over the periodic boundaries of the unit cell. The DNA was built to have the structure of a canonical double helix with a 34.28° twist per base pair, which ensured that the ends of the 42-bp fragment perfectly matched at the periodic image boundaries. All nonhydrogen atoms of the DNA were restrained harmonically to their initial coordinates with a spring constant $k_{\text{spring}}$, whose value was set to 10 kcal mol^−1^ Å^−2^ (“strong” restraints).

In total, we simulated three systems, differing by the electrolyte conditions. First, we considered the DNA surrounded by a KCl electrolyte of physiological, 0.154-M concentration, and this system was simulated under strong restraints for 125 ns. In a second system, the solvent contained a 0.0513-M solution of MgCl_2_, having the same Debye length as the 0.154-M KCl system under the Debye-Hückel approximation. This system was simulated for about 500 ns under strong restraints. Finally, we considered a fictitious system, whereby charged DNA was considered in pure water, to provide a reference for the realistic systems with electrolytes. This simulation was run for 100 ns under strong restraints.

### Preparation of the simulation systems—

The 42 base-pair DNA fragment of the poly(AT) sequence was built using the Avogadro software [63]. The molecule was solvated using the Solvate plugin of vmd [64]. Where needed, ions were added using the Autoionize plugin of vmd to first neutralize the system and then produce the desired bulk ion concentration. The required number of ions was determined from the mass ratio of water and ions, i.e., the system’s molality.

### Simulation protocols—

All MD simulations were performed using namd2.14 [65], the charmm36 parameter set [66] for protein and DNA, the TIP3P water model [67], a custom hexahydrate model for magnesium ions [30], and the CUFIX corrections to ion-nucleic acid interactions [68]. Multiple time stepping was used [69]: Local interactions were computed every 2 fs whereas long-range interactions were computed every 4 fs. All short-range nonbonded interactions were cut off starting at 1 nm and completely cut off by 1.2 nm. Long-range electrostatic interactions were evaluated using the particle-mesh Ewald method [70] computed over a 0.11-nm spaced grid. SETTLE [71] and rattle82 [72] algorithms were applied to constrain covalent bonds to hydrogen in water and in nonwater molecules, respectively. The temperature was maintained at 300 K using a Langevin thermostat with a damping constant of 0.5 ps^−1^, unless specified otherwise. Constant pressure simulations employed a Nose-Hoover Langevin piston with a period and decay of 200 and 50 fs, respectively [73]. Energy minimization was carried out using the conjugate gradients method [74]. Atomic coordinates were recorded every 9.6 picoseconds, unless specified otherwise. Visualization and analysis were performed using VMD [64] and MDAnalysis [75].

### Protocols for averaging data over the DNA base pairs—

To improve the statistical accuracy of our analysis, we averaged the data across frames of the MD trajectories and over 40 base pairs, excluding one at each end of the molecule to mitigate uncertainty arising from wrapping the solvent’s coordinates. The analysis per base pair was conducted by choosing cylindrical slabs with a radius of 30 Å, aligned along the z axis and partitioned into 40 bins. Each bin had a span of 3.4 Å, and the base pairs were positioned at the center of each cylindrical bin. This strictly geometric definition was employed to prevent double counting of atoms caused by their overlap in neighboring bins, thereby ensuring the accuracy of the calculated densities. Successive cylindrical bins were then rotated about the z axis by 34.28°, the twist per base pair for the DNA. Subsequently, the coordinates of the transformed atoms were binned on a 900 × 900 2D lattice.

### Calculation of the electrostatic potential—

The electrostatic potential was computed using the PMEpot [76] plugin of vmd. For each frame of the simulation trajectory, every point charge was approximated by a spherical Gaussian (with its inverse width referred to as the Ewald factor), normalized to give the original charge upon integration.

The instantaneous distribution of the electrostatic potential corresponding to the instantaneous charge configuration of the frame was obtained by solving the Poisson equation. The electrostatic potential maps were obtained by averaging 20–30-ns fragments of the MD trajectories. The instantaneous configurations were then averaged over the MD trajectory, taking frames every 0.25 ns. An Ewald factor of 1 Å^−1^ and 0.25 Å^−1^ was used for the fine and coarse calculations of the potential maps, respectively. The potential maps were stored as volumetric grid data with a resolution of 0.2 Å in the xy plane and about 0.98 Å along the z axis.

To obtain an average over the DNA base pairs, volumetric slices in z were rotated according to the DNA’s twist per base pair and the resolution of the grid in z, around 10.2°. For this purpose, we used the ndimage library in scipy, with a spline interpolation of order 3.

## VI. SIMULATION RESULTS

### A. Complex electrostatic environment of DNA caused by structured water

#### 1. Cylindrically averaged electrostatics of DNA

**FIG. 5.**
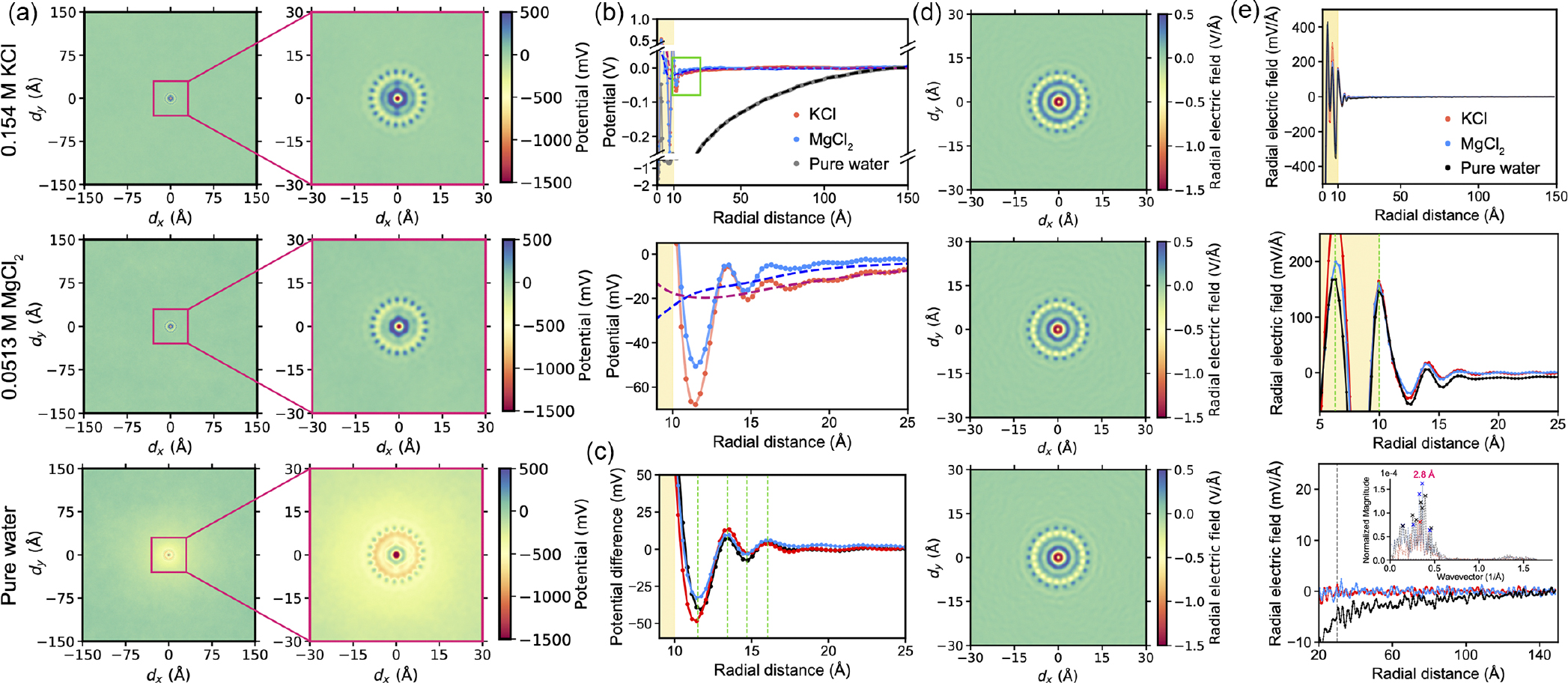
Cylindrically averaged electrostatic properties of DNA in solution: (a) Electrostatic potential maps obtained by averaging the instantaneous distributions of the electrostatic potential over the corresponding MD trajectories and along the z axis. Data for 0.154-M KCl (top) and pure water (bottom) systems were averaged over the last 100 ns of the corresponding trajectory, sampled every 2 ns. Data for the 0.0513-M MgCl_2_ system (middle) were averaged over 450 ns and sampled every 4 ns. The electrostatic potential was calculated using the VMD PMEPot plugin [76] with an Ewald factor of 1 Å^−1^. (b) Radially averaged profiles of the electrostatic potential for the KCl (red line), MgCl_2_ (blue line), and pure water (black line) systems. The bottom plot shows the same data near the DNA, whose location is indicated schematically in yellow. Solid and dashed lines correspond to the electrostatic analysis carried out using fine (Ewald factor of 1 Å^−1^) and coarse (Ewald factor of 0.258 Å^−1^) resolution. (c) Difference of the radially averaged potentials obtained using the fine and coarse electrostatic calculations. (d) Two-dimensional maps of the averaged radial electric field, calculated by locally taking the radial component of the negative gradient of the electrostatic potential map, in cylindrical coordinates. (e) Average profiles of the radial component of the electrostatic field for the three systems. The plots differ by the span of the radial distance. The region occupied by DNA is shown schematically in yellow. Dashed lines (green) indicate the locations of select peaks. The inset (bottom) shows the wave-number spectra of the averaged electric-field difference obtained through the fine and coarse electrostatic calculations. The wave-number analysis was restricted to the region 30 Å away from the DNA (dashed black line).

As a baseline for further analysis, we computed the cylindrically averaged electrostatic potential of a DNA molecule (averaged over the z axis of the simulation box) for several electrolyte conditions, similar in spirit to early analytical calculations that assumed a charged cylinder model for DNA [1,2]. Starting from a fully atomistic MD trajectory, we computed the instantaneous distributions of the electrostatic potential by solving the Poisson equation for each configuration of the partial atomic charges. In doing so, we represented each point charge of a 3D Gaussian density of the mean located at the point charge’s coordinates, the inverse width defined by the Ewald factor (i.e., 0.258 Å^−1^ for coarse and 1 Å^−1^ for fine resolution calculations), and the total integrated density equal to the point charge value. The instantaneous distributions of the electrostatic potential were averaged over the well-equilibrated parts of the respective MD trajectories and along the z axis (aligned in our coordinate system with the helical axis of the DNA) for the middle 40-base pair section of the helix. The resulting 2D densities are shown in three panels of Fig. 5(a)—for the physiological electrolyte condition (0.154-M KCl), a divalent electrolyte of the same screening length (0.0513-M MgCl_2_), and pure water.

The presence of ions in the solution produces the expected screening of the electrostatic potential, with the potential decaying away from the DNA much more rapidly in the electrolyte systems in comparison to our fictitious pure water case. The close-up views of the 2D map in the vicinity of the DNA [the right column of Fig. 5(a)] reveal concentric regions of positive and negative potential common to all three systems. While the presence of such patterns has been expected because the DNA helix itself features a regular pattern of partial charges, the presence of ions is found to profoundly modulate the magnitude and sign of the potential. Thus, in a background of negative potential due to negatively charged oxygen atoms, the 21 spots of positive potential, located approximately 10 Å away from the helix center, correspond to the positively charged phosphorous atoms of the DNA backbone, with the number of such spots reflecting the 10.5 base-pair-per-turn periodicity of the helix and the cylindrical averaging of the potential.

A feature with which we can draw direct parallels with the presented theory is that, in the presence of electrolyte ions, the inner core of DNA is found to bear a positive potential. In pure water, however, this positive core disappears, despite the potential distribution displaying a similar pattern. Guided by the theory, we can deduce that this effect must arise from the coupling of structured water dipole and ionic screening in the grooves of DNA, without either of which this phenomenon will not be present. Decomposing the electrostatic potential into its multipole expansion, a preliminary analysis shows that such a strong effect arises from the quadrupolar contribution to the interfacial water structure, a finding that is in line with previous suggestions [61]. However, given the computational bottlenecks of this analysis for a simulated system of this size, a more detailed investigation of this effect is left to future work.

Radial profiles of the electrostatic potential in the three systems provide further insight into the effect of ions on DNA electrostatics [Fig. 5(b)]. The potential at the core of the DNA (about 5 Å away from the center) is slightly higher in the KCl electrolyte than in MgCl_2_, which we attribute to the smaller hydration shells of K^+^ ions facilitating deeper partitioning of the K^+^ ions into the grooves of the DNA.

As expected, the cations of the electrolyte effectively screen the negative charge of the DNA backbone, such that the average potential approaches zero already at a distance of about 30 Å from the helix axis [Fig. 5(b), top], in contrast to the pure water system, where the potential decays down to the edge of the periodically repeated simulation box (about 150 Å).

To determine if the effect of the water structure on the DNA electrostatics can already be seen in the cylindrically averaged data, we examined the behavior of the electrostatic potential for the two electrolyte systems in the vicinity of the DNA, i.e., in the region between 9 and 25 Å from the helix axis [Fig. 5(b), bottom]. Gratifyingly, both curves reveal oscillatory modulations of the decaying potential, with the minima and maxima of the oscillations occurring at similar distances away from the helix.

To further quantify the oscillating behavior, we repeated the electrostatic calculations using a coarser Ewald factor of 0.258 Å^−1^, ensuring that the width of the Gaussian approximating each partial charge (about 3.9 Å) is larger than the size of a water molecule and expecting such a coarse approximation of the atomic charges to wash out the effect of the water structure, akin to the smearing effect described above in Sec. II and in Refs. [22,48]. Indeed, the potential curves resulting from the low-resolution electrostatic calculations did not exhibit the oscillatory pattern [Fig. 5(b), bottom]. Subtracting the low-resolution profiles from the corresponding high-resolution data isolated the effect of the water structure on the electrostatic potential [Fig. 5(c)]. We found that the minima and the maxima of the oscillations are indeed located the same distance away from the helix in the KCl and MgCl_2_ electrolytes and that the distance between the consecutive maxima are of the size of a water molecule. Repeating the low-resolution calculations for the third system, DNA in pure water, and subtracting the result from the high-resolution data, we found the oscillations of the potential were also present in the pure water system, indicating that they arise purely due to the overscreening dielectric response of water to the DNA charge [22,77,78].

Taking the negative gradient of the local electrostatic potential yielded the time-averaged electric-field vector at each voxel of each simulation system. The radial component of the electric field was then averaged over the z axis to generate the 2D maps of the electric field [Fig. 5(d)]. The resulting maps of the electric field appeared to be similar in all three systems.

The average profiles of the radial electric field elucidate the effect of ions on the local electric field [Fig. 5(e)]. Much like the electrostatic potential, the average radial component of its gradient, the electric field, displays regular alternations; however, as components of a vector quantity, they converge to oscillate around zero [Fig. 5(e), top]. As a consequence of a slightly higher potential at the core of the DNA (about 5 Å away from the center) in KCl than in MgCl_2_, the radial component of the electric field is also elevated in the KCl electrolyte, denoted by a dashed (green) line about 6.5 Å from the center [Fig. 5(e), middle], although the bulk concentrations of the systems had the same Debye length under the Debye-Hückel approximation.

Notably, at longer distances, the radial electric field still exhibits some small amplitude but persistent oscillatory patterns [Fig. 5(e), bottom]. To understand the origin of these oscillations, we analyzed the spatial Fourier transform spectra of the longer-range field (R *>* 30 Å). Similar to the electrostatic potential [Fig. 5(c)], we first subtracted the low-resolution profiles of the radial electric field from the corresponding high-resolution data, thereby isolating the effect of the water structure. The Fourier transforms of the resulting radial profiles are shown in Fig. 5(e) (bottom, inset). All three systems show dominant peaks in the wave-number spectra around 0.35 Å^−1^. The corresponding distance in real space, about 2.8 Å, is roughly the diameter of a water molecule. It is well known that in molecular simulations of water, the spatial correlation functions exhibit decaying oscillations that disappear after approximately 1–1.5 nm [79]. Thus, the coincidence of the period of these long-range oscillations may well be random, simply representing the noise, which can particularly affect the decaying tail.

**FIG. 6.**
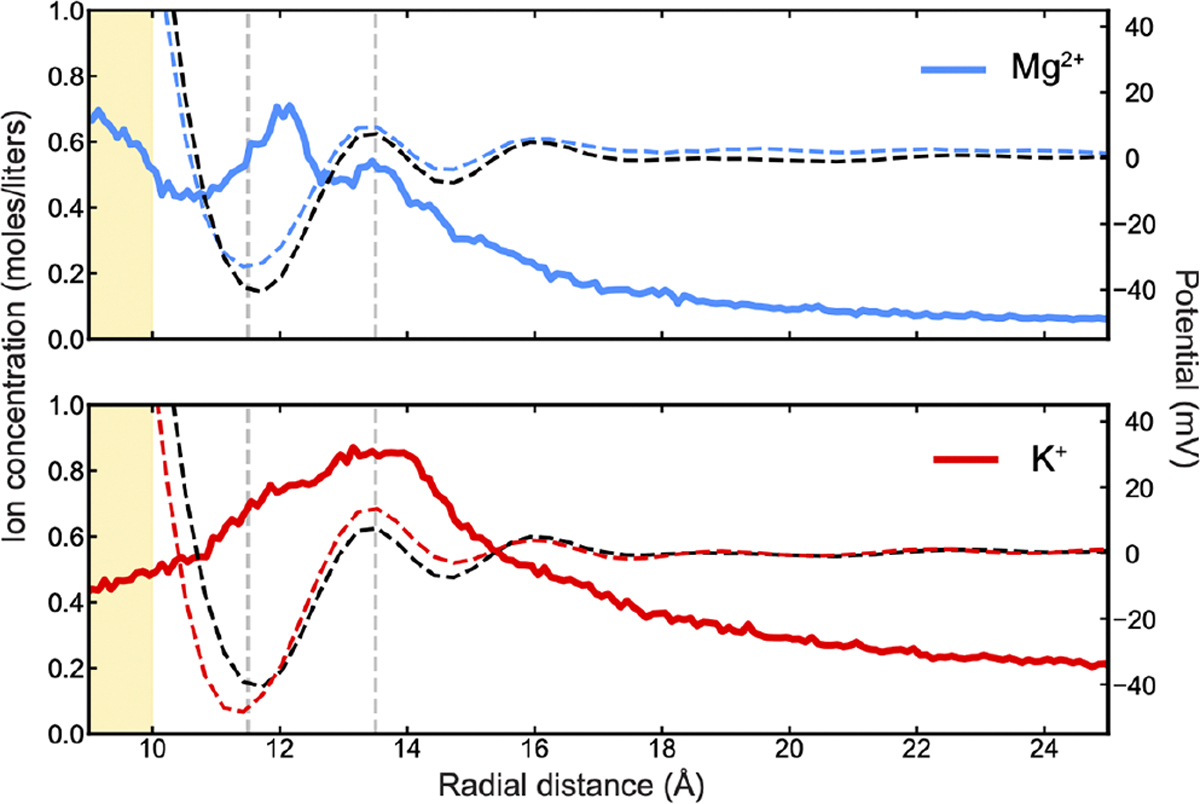
Correlation between the cation concentration and the potential difference profiles. Radial profiles of the cation densities (solid lines), for the Mg^2+^ (top) and K^+^ (bottom) systems, respectively, are plotted on the left axis. Respective profiles of the electrostatic potential difference induced by structured water are plotted on the right axis, and similarly the one for pure water is shown by the dashed line (gray). The potential difference profiles are reproduced from Fig. 5(c). The dashed vertical gray lines indicate the locations of select extremum points in the potential difference profiles, at 11.5 Å and 13.5 Å, respectively. The region occupied by DNA is schematically shown in yellow.

Bringing our attention back to the consistent large-amplitude-overscreening oscillations in the short range, the question then becomes how these oscillations influence the interactions of the DNAwith charged entities, namely, the electrolyte ions. Note that in Fig. 5(c), the oscillations of the subtracted potential in the pure water system exactly correlate with the oscillations in the electrolyte systems, which may be explained by preferential localization of the cations within the wells of the pure water potential. This hypothesis can be tested simply by superimposing the ion density profiles over the corresponding potential distributions (Fig. 6). Remarkably, in the system simulated with Mg^2+^, there is a clear peak in the ion distribution profile at around 12 Å, corresponding to a well in the potential distribution. This finding is not unexpected when considering the bulk ion concentration, as the ability for water to layer ions according to its oscillating potential distribution is largely expected in the low ion concentration regime [22], particularly for the depth of the first well. In the high-concentration regime, the ability for water to control the ion distributions is diminished as inter-ion correlations begin to dominate. Physiological concentration sits in some intermediate range between these two regimes; hence, we expected to still observe some signatures of the potential oscillations in the ion distribution profiles in our simulations with K^+^. Thus, as shown in Fig. 6, K^+^ localization is less pronounced, but there is still some indication in the ion distribution profile corresponding to this effect. In both systems, at larger distances, we see that the ions do not obey these oscillating potentials. The reason for this is clear: The depths of the wells quickly become much smaller than $k_{B}T/e$, so they are not strong enough to fight the entropic urge for ions to spread around.

Both the profiles of ion density and of the electrostatic potential caused by DNA, in pure water and in electrolyte solution, display some signatures of decaying oscillations. Such patterns were previously studied near flat surfaces in the context of electrochemical double layer [17–21], ITIES [80], and near lipid membranes [81], as well as charged mica surfaces [82], and all these effects were discussed in a recent review [83]. Similar signatures in water near DNA indicate the ubiquitous manifestation of the water structure at the nanoscale. It should be stressed, again, how the ion-specific interactions with the DNA were considered. In the molecular simulations, the force field specifies how ions interact with water and nucleotides, effectively prescribing the pattern of ion localization near the DNA [68]. In the theory, such interactions are accounted for phenomenologically, treating ions that specifically adsorb into the DNA grooves as part of the overall DNA charge pattern.

#### 2. DNA electrostatics in the reference frame of a DNA base pair

**FIG. 7.**
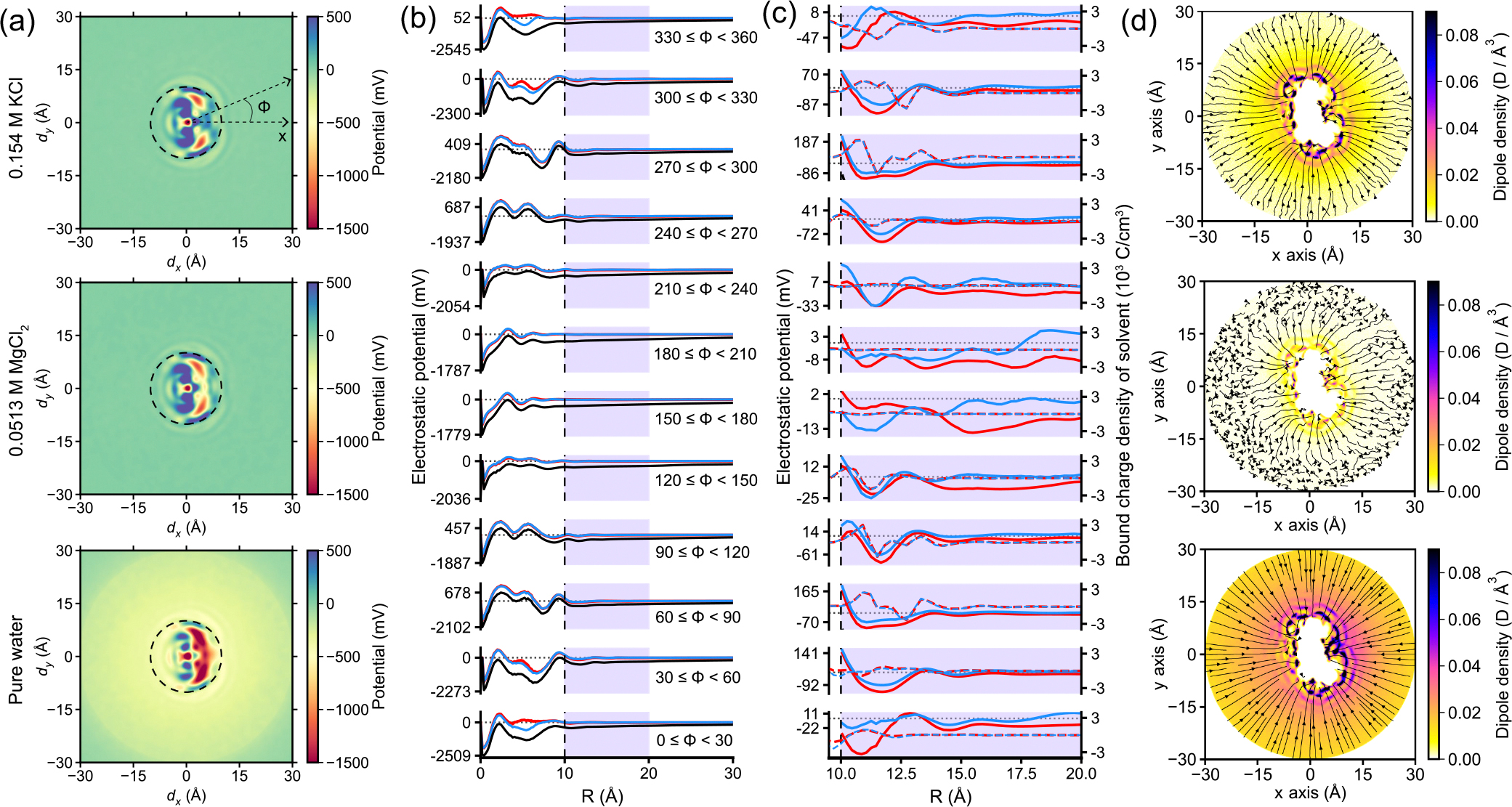
Base-pair-reference averaged electrostatic properties of DNA in solution. (a) Projected 2D maps of the electrostatic potential averaged upon alignment of individual base pairs to the reference, for 0.154-M KCl (top), 0.0513-M MgCl_2_ (middle), and the pure water system (bottom). Excluding the base pair on both ends, the average is based on the last 100 ns for KCl and pure water, and the last 450 ns for MgCl_2_. The trajectory coordinates were analyzed every 2 ns for the KCl and pure water systems or every 4 ns for the MgCl_2_ one. (b) Radial profiles representing consecutive angular patches measured with respect to the *x* axis, illustrated by the schematic in the top of panel (a). Potential profiles were generated by averaging over voxels within consecutive radial shells of the respective angular patches. Line colors follow the same convention as in Fig. 5, with K^+^ shown in red, Mg^2+^ in blue, and pure water in black. The angular range corresponding to each subplot is indicated below the respective curve. (c) Zoomed-in view of the shaded (purple) patch in panel (b) (10 *<* R *<* 20 Å). The right axis (dashed lines) is the total charge density averaged over the respective angular patches, for K^+^ (red) and Mg^2+^ (blue). The pure water lines are not visible at this scale. The dotted (black) line is a guide to the eye, marking zero for both left and right axes. (d) Dipole moment originating from water molecules. Colors in the 2D map represent the local dipole moment density, whereas the black lines depict the average direction interpolated over the nearby voxels. Any dipole moment arising from separation of ions has been neglected in the analysis of the electrolyte systems.

Subsequently, we investigated the average electrostatic properties surrounding the DNA base pair (Fig. 7). To enhance statistical accuracy, individual slabs of the 3D potential map were aligned with respect to the reference base pair and then averaged (see Sec. V for details). With added electrolytes, the potential profiles for K^+^ and Mg^2+^ systems decay faster, as expected, with the 2D potential maps displaying striking similarities [Fig. 7(a), top and middle].

Notably, these characteristics are also somewhat evident in the fictitious pure water system, albeit, as it should be, with significantly reduced screening levels [Fig. 7(a), bottom]. Corresponding line plots compare these systems on a common scale [Fig. 7(b)]. Inside the DNA core, 0<R<10Å, the electrostatic potential profiles follow similar trends in all three systems, with a weaker, dielectric screening in the case of pure water. In the region just outside the DNA core but in close proximity, 10<R<20Å, the oscillations are much less pronounced [Fig. 7(c)].

In Fig. 7(c), the patterns of oscillations of the electrostatic potential in the presence of K^+^ (red) and Mg^2+^ (blue), respectively, are very similar, with subtle, minor differences depending on the azimuthal direction. The solvent bound charge density is plotted [Fig. 7(c), right axis] with the potential, revealing a close correlation between them. Minor distinctions between those patterns arise, possibly as a consequence of the nonlinear dielectric response of the electrolyte solution to the nearby phosphate groups. Nonetheless, the overarching patterns exhibit notable correlations.

The uniform characteristics noted in the potential maps across all three systems, irrespective of the presence of electrolytes, suggest that the preferential alignment of water molecule dipoles may play a pivotal role, particularly so in the absence of any electrolyte. We explicitly verify this hypothesis by tracking the precise locations of water molecules around the DNA. As shown in Fig. 7(d), it is evident that water dipoles arrange themselves to minimize the potential energy surface near the DNA molecule. The introduction of cations compensates for the entropic cost linked with the precise localization of water dipoles. A comparison between results for the “pure water system” (bottom) and the electrolytic solution (top and middle) reveals higher-dipole-moment magnitudes, and the interpolated dipole vectors exhibiting a striking parallel alignment. As expected, the degree of ordering is lower in the presence of Mg^2+^ ions than in K^+^. In Mg^2+^, the ordering is almost gone around 20 Å from the DNA axis.

#### 3. Long-range screening

**FIG. 8.**
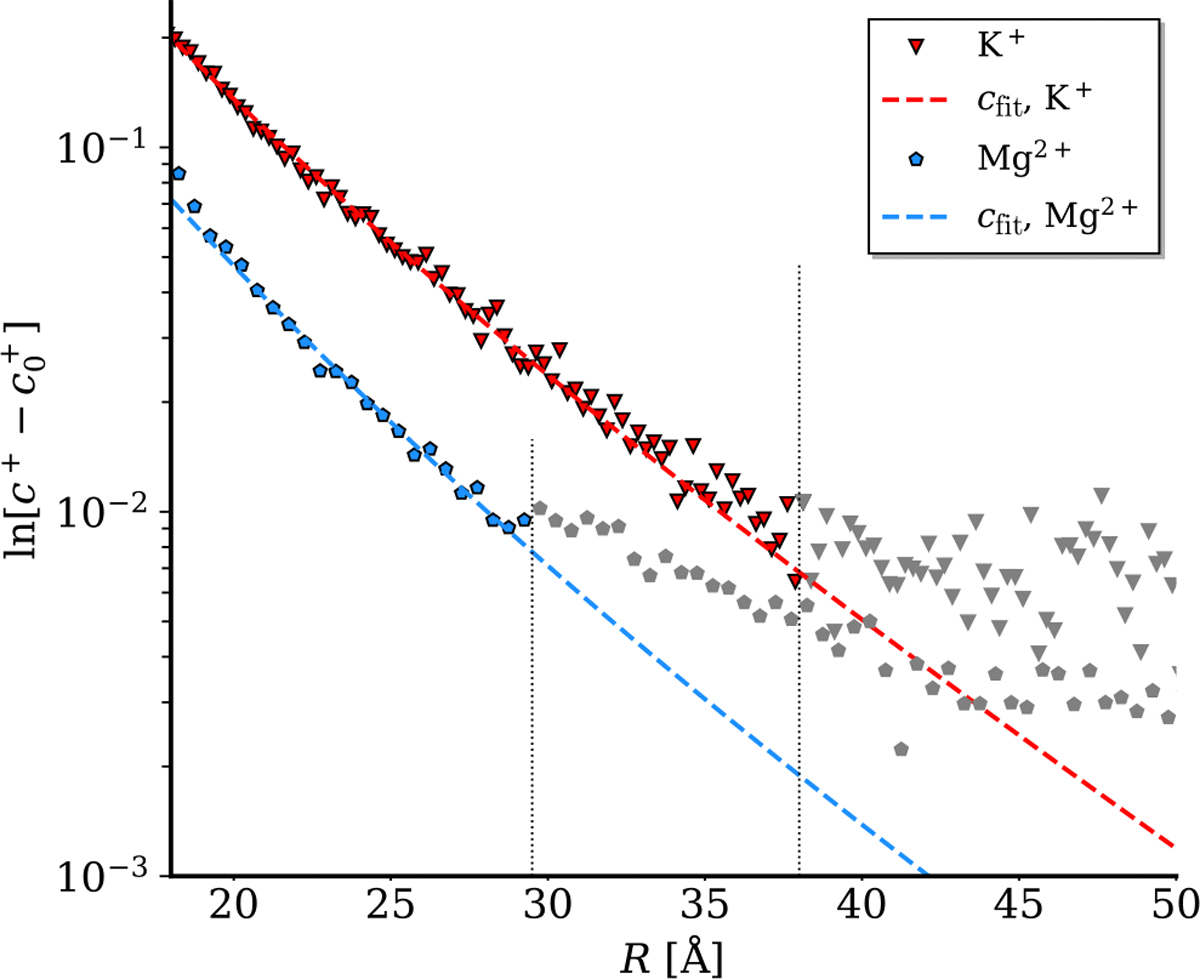
Long-range electrostatic screening in MD simulations. Radially averaged profiles of K^+^ and Mg^2+^ concentrations, showing two screening regimes in the electrostatics of DNA. The profiles are fitted using Eq. (46), and the resulting fits are shown as dashed lines. The fit is remarkably accurate in the intermediate range, where the screening behavior is characterized by the “renormalized Debye length,” $\lambda_{D}=1/\sqrt{\kappa^{2}+g^{2}}$. As reasoned in the text, the reproduction of longer-range Debye screening would require unrealistically large simulation volume and simulation times. The data that might be affected by the finite-size effects (after the transitions between regimes denoted by the vertical dotted lines) are shown in gray. The fitting prefactors are calculated as follows: (i) for K^+^, A=7.068,B=1.528; (ii) for Mg^2+^, A=3.624 and B=0.389.

The relatively large size of our MD systems allows us to investigate the long-range electrostatic behavior, namely, the Debye decay tail. As mentioned in the theory section, the long-range electrostatic behavior is important, as it dictates the forces felt by other charged species in solution, which are key to the vast majority of biological processes and interactions [84]. It should be noted that examining the long-range behavior may only be possible in very long simulations of large systems at high ion concentrations [85] and may require numerical corrections [86]. As our simulations were performed at physiological concentrations, we did not expect to find an accurate recapitulation of the theoretical results. Yet, we expected to see distinct screening behaviors, enabling comparison with the predictions of the theory. Instead of analyzing the electrostatic potential as done in the theory, the ion distribution can be used here as a proxy, given that it shows much less fluctuation in the long range [87]. Additionally, within the linearized Poisson-Boltzmann approximation, there is a linear relationship between the potential and ionic charge density (relative to the bulk). This approximation is expected to hold at larger distances (R>20Å) from the charged DNA surface, so both should provide equivalent information about the modes of screening. Indeed, we see this linear relationship in the simulation results in Fig. 8 by plotting the concentration profiles of both K^+^ and Mg^2+^ on a logarithmic scale. Much closer to the DNA, for 10<R<20Å, such a relationship between the ion densities and the electrostatic potential may break down. These concentration profiles shown in Fig. 8 reveal the presence of two screening regimes, with a transition between them occurring at some intermediate distance from the DNA surface, in line with theoretical predictions. As reasoned above, these ion concentration profiles are still roughly proportional to the potential, so a simple fitting function can be written to describe them, inspired by Eqs. (38)–(42),

(46) $$c_{\text{fit}}(R)=AK_{1}\left({\tilde{\kappa }}_{1}R\right)+BK_{0}(\kappa R).$$

To restate, $K_{n}(x)$ is the nth order modified Bessel function of the second kind, ${\tilde{\kappa }}_{1}=\sqrt{\kappa^{2}+g^{2}}$, and g=2π/H, where H≈34Å is the helical pitch of the DNA molecule. The prefactors A and B are fitting parameters; within the theory, the balance between these parameters will depend on a complex relationship between the counterion adsorption fractions $f_{i}$ and the overall counterion compensation Θ, which in turn will depend on the specific electrolyte ions we wish to examine. Fitting the data, we focus on the intermediate region 20<R<40Å. Remarkably, we indeed see the presence of this renormalized Debye screening region in both K^+^ and Mg^2+^ electrolyte simulations, where the Debye length of the electrolyte $\kappa^{-1}$ is renormalized by the helical structure of the DNA to become $\lambda_{D}\approx 1/\sqrt{\kappa^{2}+g^{2}}$. For simulations performed with K^+^, this screening regime persists until R∼38Å, which agrees well with the parameters used for the plots in Figs. 3 and 4. However, this transition occurs at closer distances of R∼29-30Å in the Mg^2+^ case, as indicated by the dashed line in Fig. 8.

The quantitative difference in the behavior of the two electrolytes is understandable when we consider the effect of counterion condensation on the DNA molecule. The location of the transition to renormalized screening length depends on the balance of the fitting prefactors A and B, which are functions of the counterion adsorption pattern fractions and the overall charge compensation. Studies examining the adsorption of K^+^ and Mg^2+^ to DNA suggest that the two ionic species have similar adsorption patterns, both preferentially binding to the major groove [34], thus leaving the charge compensation Θ to be the only strong remaining factor in this analysis. Naturally, the divalency of the Mg^2+^ ions will lead to much stronger charge compensation and hence nontrivial suppression of the different helical harmonics, causing the shift in the cross-over point. Note that this behavior may not be reflected in the fitting parameters because of the inaccuracy associated with modeling the longer-range screening behavior.

It is important to also note that, in principle, the present linear electrostatic model of the system was not expected to hold for Mg^2+^ at such bulk concentrations. It is well known that the high charge density of the ions leads to strong nonlinearity in their electrostatics. However, as a result of our simulations, it seems that, also at these physiological concentrations (and hence larger Debye lengths), linear electrostatics can be a valid approximation for biological systems, contrary to the previous arguments [3,15].

## VII. DISCUSSION AND CONCLUSIONS

In this work, we have conducted an in-depth joint theoretical and computational study of the electrostatics of DNA. Taking into account all the complexities of the system, namely, the double helicity of the DNA molecule, finite size, charge fluctuations through smearing effects, and, most importantly for this paper, the nonlocal dielectric properties of the solvent, we were able to construct a theoretical framework in which these electrostatics can be analyzed.

These theoretical results and predictions have been substantiated by extensive computational characterization through all-atom molecular dynamics simulations, where we have analyzed and observed, to high accuracy, this complex interplay between the DNA and the solvent and electrolyte ions. Given the number of plots shown in each panel of Figs. 3–8, we presented the results of the theory and simulations separately. Appendix D presents a direct comparison, showing the differences in these results as line plots on the same set of axes. We comment there on discrepancies between the linear response theory and the simulations, the latter of which may contain nonlinear, dielectric saturation effects [49,88], which we also discuss in Appendix D.

### Key points—

Through both theoretical and computational analyses we were able to understand the following:

- The electrostatic potential inside the DNA is positive, despite having such a strong negative charge from the phosphate backbone. Such an effect results from the coupled screening effects of structured water in the grooves and ion localization.
- There are strong electric-field oscillations in the near vicinity of the DNA molecule. Analysis of the polarization density of water shows that these oscillations arise from structured water, both within the grooves of DNA and on the DNA phosphate backbones.
- At physiological concentrations of KCl, the presence of ions does not disrupt these oscillations, which is evident from comparing both the polarization density [Fig. 7(d)] and the electrostatic potential profiles [Figs. 5(b) and 7(b)] in both pure water and physiological electrolyte. Rather, the ions simply screen the potential, shifting the profile in the positive direction, and they only slightly affect the structuring of the water dipoles. This result for the electrostatic potential is also observed for MgCl_2_, although at the concentration considered, the cations disrupt this water structuring [Fig. 7(d), middle] more strongly.
- The electric-field distribution across simulations shows little to no difference in the presence or absence of cations, a result that is mirrored by the theoretical model. Such a consistent result across simulations implies that, in the close range, water dominates the electrostatics of a DNA molecule at physiological concentrations, validating the results of the theory.
- We can see that the first layer of ions in the double layer prefers to be localized in the first potential well created by the overscreening dielectric response of water to the DNA charge. In other words, these ions are physisorbed preferentially with their first hydration shell. Further away from the DNA, these trends are less obvious due to the diminished depth of the wells.
- At intermediate distances, outside of the range of water correlations, the Debye length is renormalized, i.e., effectively decreased, by coupling to the double-helical structure of the DNA molecule.
- The Lorentzian contribution to the dielectric response of water, which effectively reduces the dielectric constant close to the DNA surface, dramatically enhances the electrostatic potential and the electric field in that range, becoming unimportant at long distances.

### Points to consider–

Despite the good agreement between the simulations and theory, it is important to note the approximations used within the theory. First, we do not account for the excluded volume of the DNA itself, as per the embedded charge approximation (see Sec. II B 1.). Hence, the results obtained within one molecular diameter of water, about 2.5 Å around each helical line, must be taken with caution. This approximation together with the overscreening dielectric response of water, gives rise to an artefact: the sign of the potential is inverted at these charged helical lines. Smearing of these lines diminishes this artefact, but does not remove it completely. Second, the approximation used for the dielectric function of the electrolyte is the simplest “interpolation” form we can use. Such a form allows analytical calculation of the electrostatic potential, leading to reasonable results as compared to MD simulations. Still, it must be acknowledged that the quantitative results will depend strongly on the model of dielectric response used.

The effect of undulations within the primitive model of the solvent was studied earlier in Ref. [89]. It was found to be substantial in the interaction between long DNA molecules in dense DNA fibers. However, again, undulations have wavelength of the order of bending persistence length 500 Å, much larger than all the characteristic scales of the structure of water. The same is true for the torsional fluctuations [90,91]. In the theory, thermal fluctuations of the individual charge groups were taken into account here through introduction of Debye-Waller-like factors, and in the simulations, they were controlled by bond-simulating potentials in the force fields, plus an additional restraining potential that ensured that the DNA axis was kept straight.

To understand the role of the latter, for comparison, we also performed simulations with a much softer restraining potential ($k_{\text{spring}}=0.5\;kcal\;{mol}^{-1}{\;Å}^{-2}$ applied on every nonhydrogen atom). We see some quantitative effects, which are discussed in Appendix C.

Note that in the theory, we modeled the DNA molecule as an ideal double helix, with the intention to unravel the interplay between the corresponding pattern of the DNA charge distribution and the structure of water in the formation of the electric field. Understanding this case is a necessary and natural first approach to the problem. To be consistent with this approach, simulations were performed for a specific sequence in which the distortions of the helical structure were minimal. This sequence consisted of two single strands—one poly(AT) and one poly(TA)—hybridized in a double-helical structure with minimal variance of twist and rise.

Earlier theoretical approaches have investigated the effect of sequence-dependent nonideal helicity leading to accumulation of helical distortions over distances larger than the so-called helical coherence length $\lambda_{c}$ [12]. This effect is important in the interaction of long DNA tracts, longer than $\lambda_{c}$ [12,92–94], estimated from experiments as 11 nm [95]. Thus, as the range of correlations of polarization fluctuations in water (of the order of 1 nm) are much shorter than $\lambda_{c}$, such distortions are not expected to affect the distribution of the local electrostatic field around DNA. However, this idea does not dismiss future, more-detailed investigations of this issue, although we do not anticipate any new significant qualitative changes.

However, given the good agreement with simulations, we are confident that this analytical, linear, nonlocal electrostatic theory captures the key features of the electric field around DNA and hence emphasizes the importance of the solvent structural effects in the electrostatics of this most important molecule of life.

## Figures and Tables

**FIG. 9.**
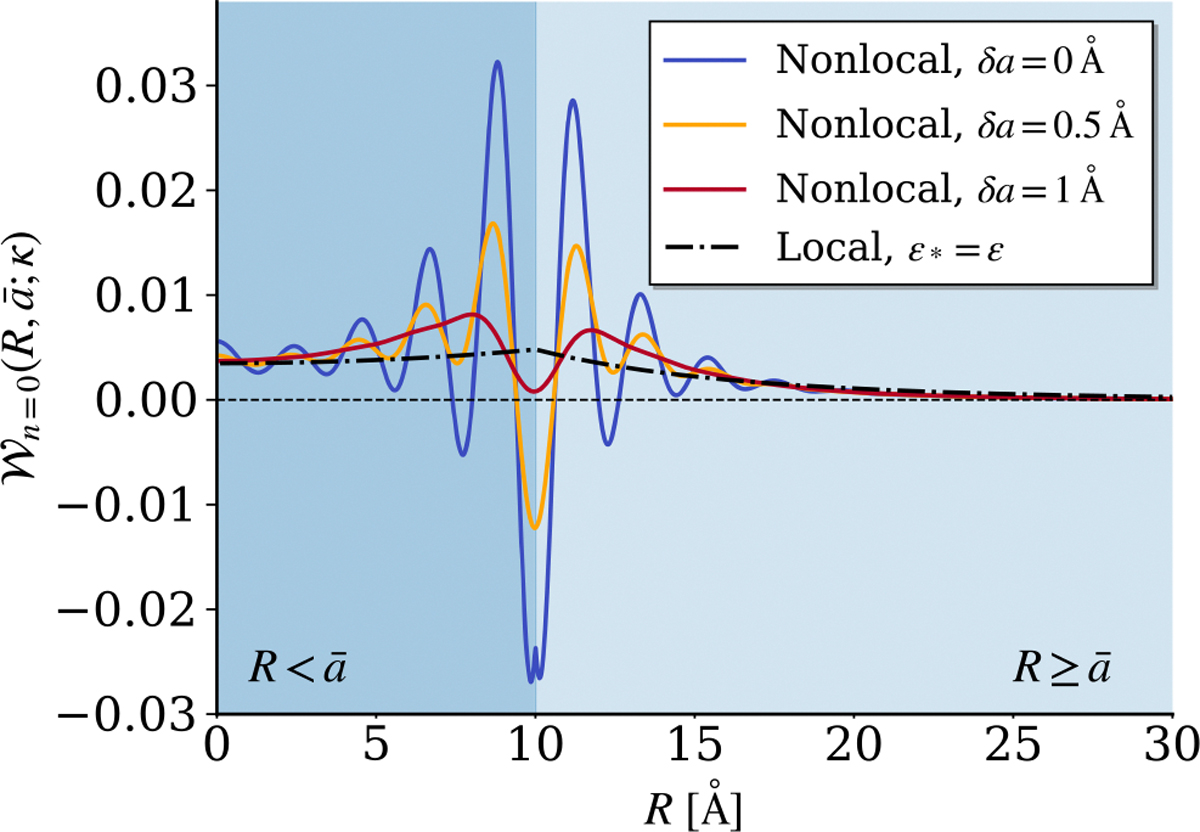
Comparison of nonlocal and local calculations of the electrostatic propagator, and the effect of smearing. Here, the zeroth harmonic (n=0) of Eq. (42) is plotted, with the characteristic lengths and coefficients defined as in Eqs. (A1)–(A7). The nonlocal parameters and Debye length are as defined in Fig. 3. The local limit is obtained by setting $\varepsilon_{*}=\varepsilon$. Smearing of the electrostatic potential is performed by applying the smearing operator $\overset{ˆ}{\zeta }$, as defined in Eq. (45), with $\overset{‾}{a}=10\;Å$, for the smearing parameters δa, indicated in the figure legend.

**FIG. 10.**
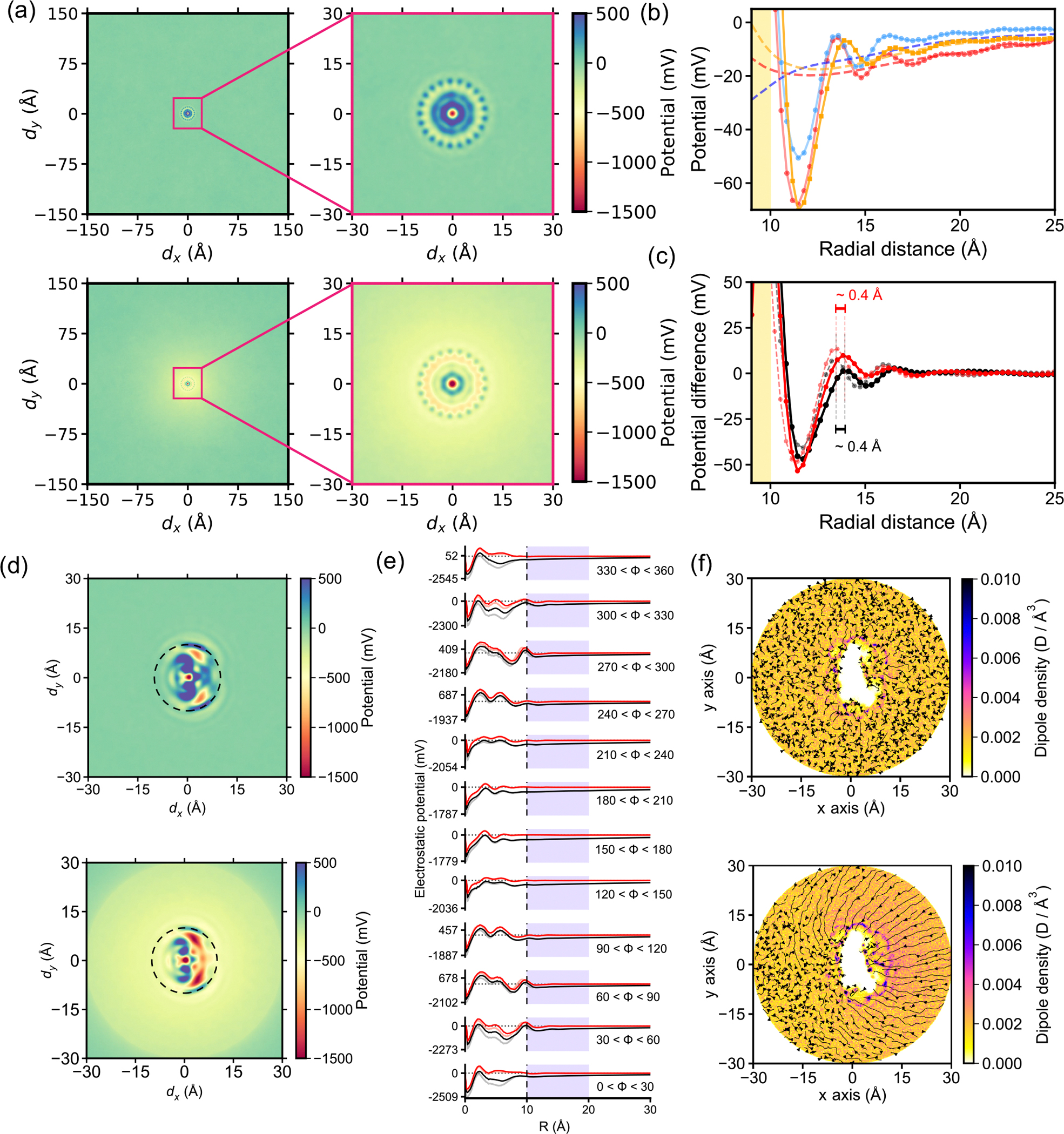
Effect of restraining the potential on the MD description of DNA electrostatics. (a) Cylindrically averaged electrostatic potential maps for KCl (top) and pure water (bottom) systems. (b) Radially averaged profiles of the electrostatic potential for KCl (red) and MgCl_2_ (blue) with the stiff spring ($k_{\text{spring}}=10\;kcal{\;mol}^{-1}{\;Å}^{-2}$), and KCl (orange) with the weaker spring ($k_{\text{spring}}=0.5\;kcal{\;mol}^{-1}\;{Å}^{-2}$). Solid and dashed lines have the same meaning as in Fig. 5(b), bottom. (c) Difference potentials computed as in Fig. 5(c). KCl (red) and pure water (black) systems with the stiff spring are made transparent. (d) Base-pair-reference averaged electrostatic potential map for KCl (top) and pure water (bottom). (e) Radial profiles representing consecutive angular patches measured with respect to the x axis. The analysis and line colors follow the same convention as in Fig. 7(b). The systems with the stiff spring are shown as light gray lines. (f) Dipole moment originating from water molecules, for KCl (top) and pure water (bottom) with the weaker spring $k_{\text{spring}}=0.5\;kcal{\;mol}^{-1}{\;Å}^{-2}$. Equivalent plots for the stiff spring are shown in Fig. 7(d). The analysis procedure and plot characteristics follow the same convention as in Fig. 7(d).

**FIG. 11.**
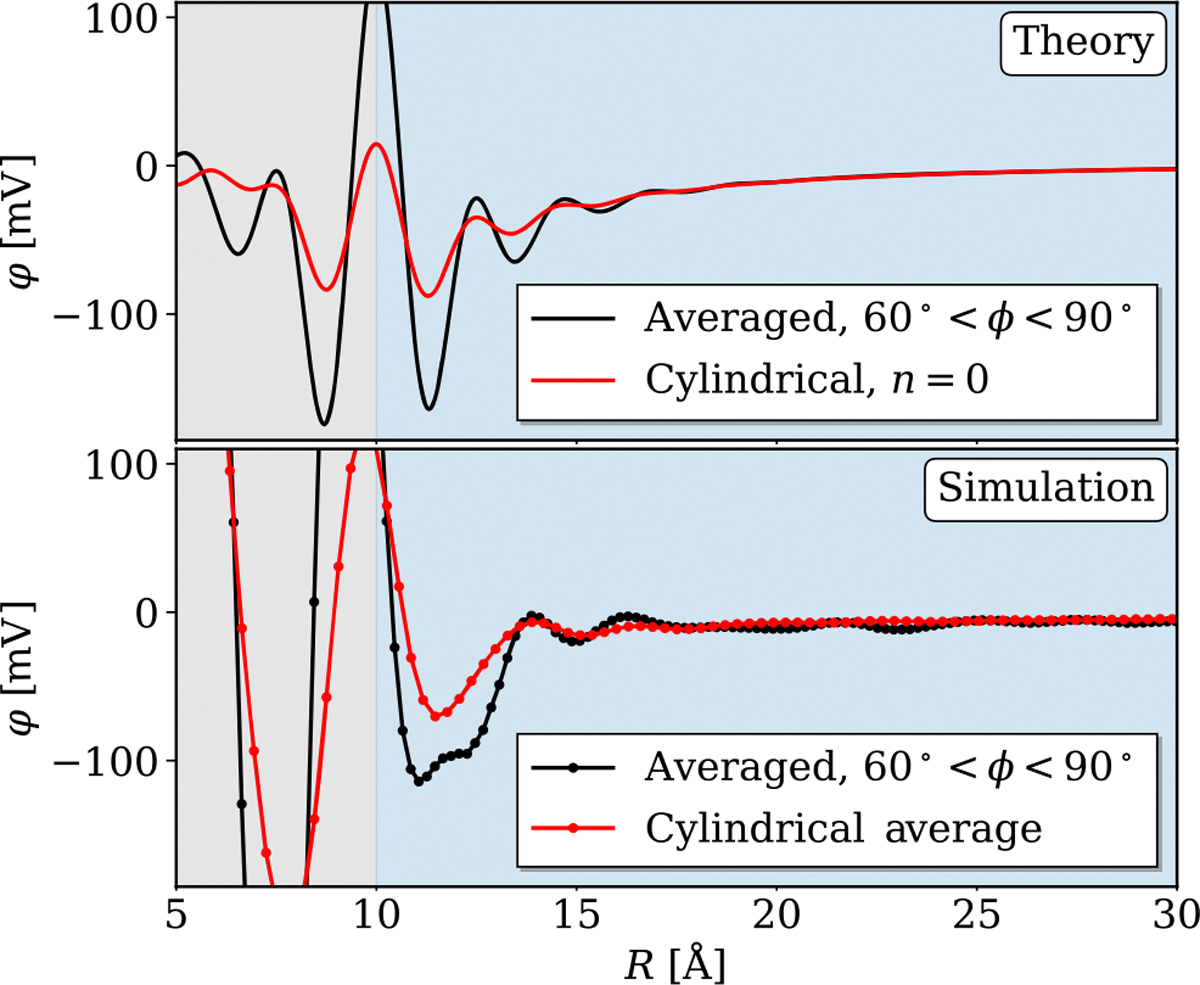
Direct comparison of the electrostatic potential between the theoretical model and molecular dynamics simulations. Line plots of the electrostatic potential as a function of radial distance R are shown as calculated in the theory [Eqs. (38)–(42)], with parameters taken as in Fig. 3 (top) and atomistic molecular dynamics simulations (bottom). The black lines show the potential averaged within a 30° window, chosen such that it encompasses one phosphate group. The red lines show a cylindrical average, which, in the theory, corresponds to the n=0 term in Eqs. (38)–(42).

## APPENDIX A: NONLOCAL SPATIAL ELECTROSTATIC PROPAGATOR

### 1. Characteristic lengths and coefficients

Equation (42) contains a number of parameters that were too cumbersome to include in the main text. Their expressions result from taking the integral over K in Eq. (24) by contour integration, with the dielectric function defined as in Eqs. (6) and (7). We present these expressions here, in terms of the quantities defined in the main text.

(A1)
$${\tilde{\kappa }}_{n}=\sqrt{\kappa^{2}+n^{2}g^{2},}$$

(A2)
$${\tilde{q}}_{d1}^{n}=\sqrt{\frac{1}{\Lambda_{1}^{2}}+n^{2}g^{2}},$$

(A3)
$${\tilde{g}}_{n}^{R}=\sqrt{\frac{Q^{2}\;-\;q_{d2}^{2}\;-\;n^{2}g^{2}\;+\;\sqrt{{\left(Q^{2}\;-\;q_{d2}^{2}\;-\;n^{2}g^{2}\right)}^{2}\;+\;4Q^{2}q_{d2}^{2}}}{2}},$$

(A4)
$${\tilde{g}}_{n}^{I}=\sqrt{\frac{q_{d2}^{2}\;-\;Q^{2}\;+\;n^{2}g^{2}\;+\;\sqrt{{\left(Q^{2}\;-\;q_{d2}^{2}\;-\;n^{2}g^{2}\right)}^{2}\;+\;4Q^{2}q_{d2}^{2}}}{2}},$$

where we can clearly see that ${\tilde{g}}_{n}^{R}\to Q$ and ${\tilde{g}}_{n}^{I}\to q_{d2}=1/\Lambda_{2}$ for n→0, as stated in the main text. The prefactors for each term in Eq. (42) are given by

(A5)
$${\tilde{g}}_{\kappa }=\frac{1}{\varepsilon_{*}}-\frac{\gamma }{1\;-\;\kappa^{2}\Lambda_{1}^{2}}\left(\frac{1}{\varepsilon_{*}}\;-\;\frac{1}{\varepsilon }\right)\;\;-\frac{(1\;-\;\gamma ){\left(q_{d2}^{2}\;+\;Q^{2}\right)}^{2}}{Q^{4}\;+\;2Q^{2}\left(q_{d2}^{2}\;+\;\kappa^{2}\right)\;+\;{\left(q_{d2}^{2}\;-\;\kappa^{2}\right)}^{2}}\left(\frac{1}{\varepsilon_{*}}\;-\;\frac{1}{\varepsilon }\right),$$

(A6)
$${\tilde{g}}_{L}=\frac{1}{1\;-\;\kappa^{2}\Lambda_{1}^{2}}\left(\frac{1}{\varepsilon_{*}}-\frac{1}{\varepsilon }\right),$$

(A7)
$${\tilde{g}}_{O}=\frac{\pi }{4}\frac{{\left(q_{d2}^{2}\;+\;Q^{2}\right)}^{2}}{Qq_{d2}\left(Q^{2}\;-\;q_{d2}^{2}\;-\;2iQq_{d2}\;+\;\kappa^{2}\right)}\left(\frac{1}{\varepsilon_{*}}-\frac{1}{\varepsilon }\right).$$

### 2. Comparison with local electrostatic approximation

While the structure and terms of Eq. (42) were discussed in Sec. III C, it is not immediately clear how the electrostatic potential will differ from the classical, local theory with the combination of Bessel and Hankel functions presented. The classical limit can be obtained rather simply by considering $\varepsilon_{*}\to \varepsilon$, which, from Eq. (6), leads to a macroscopic dielectric response, where ε(k)=ε. This result can clearly be seen in the coefficients in Eqs. (A5)–(A7), where the nonlocal “correction” terms go to zero in the local limit.

In Fig. 9, we show a comparison of calculations of the electrostatic propagator, based on the nonlocal theory, Eq. (42), with the results of a calculation in which the dielectric response of the solvent is described by a constant. Note here that only the zeroth harmonic (n=0) of the propagator is plotted, which is proportional to the electrostatic potential created by a homogeneously charged cylinder. As discussed for a simple spherical ion in Sec. II B, the water structure manifests as decaying oscillations, a result of the “overscreening” dielectric response of water. When compared to the local limit, the electrostatic potential is greatly enhanced in the vicinity of the charge distribution.

However, for DNA in reality, oscillations of such large amplitudes will never emerge. Indeed, thermal fluctuations and the excluded volume of the charge distribution will substantially suppress these oscillations. These effects can be accounted for in the simplest way by smearing of the charge distribution in the radial direction, which is done by application of the smearing operator $\overset{ˆ}{\zeta }$, as defined in Eq. (45). As expected, for intermediate levels of smearing, the amplitudes of the oscillations are dramatically reduced, yet they still show a significant deviation from the local limit. For larger smearing parameters, the oscillatory patterns are entirely dephased. However, effects of nonlocality still remain; there is an enhancement in the profile close to the charge, a result of the Lorentzian contribution to the dielectric response.

## APPENDIX B: ANALYTICAL EXPRESSIONS FOR ELECTRIC FIELD AND CHARGE DENSITY

Here, we present analytical expressions for the electric field E and the total charge density ϱ derived from the electrostatic potential. In cylindrical coordinates,

(B1)
$$E=-\left\{\frac{\partial }{\partial R}u_{R}+\frac{1}{R}\frac{\partial }{\partial \phi }u_{\phi }+\frac{\partial }{\partial z}u_{z}\right\}\varphi ,$$

where $u_{R},\;u_{\phi }$, and $u_{z}$ are the unit vectors of the three components of the electric field. Each of these components can be split into three contributions: DNA, phosphate charges of DNA; CC, condensed counterions; and W, the surface bound charge.

(B2)
$$E_{R}^{DNA}=-8\pi a_{DNA}\overset{‾}{\sigma }\sum_{n=0}^{\infty }\;\frac{e^{-\frac{1}{2}n^{2}g^{2}\Delta_{eff}^{2}}}{\delta_{n,0}\;+\;1}cos[n(\phi -gz)]cos\left[\frac{n\phi_{s}}{2}\right]{𝒲}_{n}^{\prime }\left(R;a_{DNA},\kappa \right),$$

(B3)
$$E_{z}^{DNA}=-8\pi a_{DNA}\overset{‾}{\sigma }\sum_{n=1}^{\infty }\;nge^{-\frac{1}{2}n^{2}g^{2}\delta z_{str}^{2}}sin[n(\phi -gz)]cos\left[\frac{n\phi_{s}}{2}\right]{𝒲}_{n}\left(R;a_{DNA},\kappa \right),$$

(B4)
$$E_{\phi }^{DNA}=\frac{8\pi a_{DNA}\overset{‾}{\sigma }}{R}\sum_{n=1}^{\infty }\;ne^{-\frac{1}{2}n^{2}g^{2}\delta z_{str}^{2}}sin[n(\phi -gz)]cos\left[\frac{n\phi_{s}}{2}\right]{𝒲}_{n}\left(R;a_{DNA},\kappa \right),$$

(B5)
$$E_{R}^{CC}=8\pi a_{CC}\overset{‾}{\sigma }\Theta \sum_{n=0}^{\infty }\;\frac{\left(f_{1}e^{-\frac{1}{2}n^{2}g^{2}\delta z_{c1}^{2}}\;+\;f_{2}(-1{)}^{n}e^{-\frac{1}{2}n^{2}g^{2}\delta z_{c2}^{2}}\right)}{\delta_{n,0}\;+\;1}cos[n(\phi -gz)]{𝒲}_{n}^{\prime }\left(R;a_{CC},\kappa \right),$$

(B6)
$$E_{z}^{CC}=8\pi a_{CC}\overset{‾}{\sigma }\Theta \sum_{n=1}^{\infty }\;ng\left(f_{1}e^{-\frac{1}{2}n^{2}g^{2}\delta z_{c1}^{2}}+f_{2}(-1{)}^{n}e^{-\frac{1}{2}n^{2}g^{2}\delta z_{c2}^{2}}\right)sin[n(\phi -gz)]{𝒲}_{n}\left(R;a_{CC},\kappa \right),$$

(B7)
$$E_{\phi }^{CC}=-\frac{8\pi a_{CC}\overset{‾}{\sigma }\Theta }{R}\sum_{n=1}^{\infty }\;n\left(f_{1}e^{-\frac{1}{2}n^{2}g^{2}\delta z_{c1}^{2}}+f_{2}(-1{)}^{n}e^{-\frac{1}{2}n^{2}g^{2}\delta z_{c2}^{2}}\right)sin[n(\phi -gz)]{𝒲}_{n}\left(R;a_{CC},\kappa \right),$$

(B8)
$$E_{R}^{W}=8\pi a_{W}{\overset{‾}{P}}_{0}\sum_{n=0}^{\infty }\;\frac{\left(w_{1}e^{-\frac{1}{2}n^{2}g^{2}\delta z_{w1}^{2}}\;+\;w_{2}(-1{)}^{n}e^{-\frac{1}{2}n^{2}g^{2}\delta z_{w2}^{2}}\right)}{\delta_{n,0}+1}cos[n(\phi -gz)]{𝒲}_{n}^{\prime }\left(R;a_{W},\kappa \right),$$

(B9)
$$E_{z}^{W}=8\pi a_{W}{\overset{‾}{P}}_{0}\sum_{n=1}^{\infty }\;ng\left(w_{1}e^{-\frac{1}{2}n^{2}g^{2}\delta z_{w1}^{2}}+w_{2}(-1{)}^{n}e^{-\frac{1}{2}n^{2}g^{2}\delta z_{w2}^{2}}\right)sin[n(\phi -gz)]{𝒲}_{n}\left(R;a_{W},\kappa \right),$$

(B10)
$$E_{\phi }^{W}=-\frac{8\pi a_{W}{\overset{‾}{P}}_{0}}{R}\sum_{n=1}^{\infty }\;n\left(w_{1}e^{-\frac{1}{2}n^{2}g^{2}\delta z_{w1}^{2}}+w_{2}(-1{)}^{n}e^{-\frac{1}{2}n^{2}g^{2}\delta z_{w2}^{2}}\right)sin[n(\phi -gz)]{𝒲}_{n}\left(R;a_{W},\kappa \right),$$

where, in Eqs. (B2), (B5), and (B8), ${𝒲}_{n}^{\prime }=\partial {𝒲}_{n}/\partial R$.

To incorporate the effect of smearing of the charge distributions, each of these contributions will be smeared with the operator ${\overset{ˆ}{\zeta }}_{\nu }$ as defined in the main text. Each component of the electric field will then be ${\overset{‾}{E}}_{\beta }^{\text{tot}}=\sum_{\nu }{\overset{ˆ}{\zeta }}_{\nu }E_{\beta }^{\nu }$ (the bar signifies smearing), where β={R,ϕ,z} and ν={DNA,CC,W}. Thus, the magnitude of the smeared electric field is

(B11)
$$\left|{\bar{E}}_{\text{total}}\right|=\sqrt{\sum_{\beta }\;{\left({\overset{‾}{E}}_{\beta }^{tot}\right)}^{2}}.$$

The total charge density is proportional to the Laplacian of the potential,

(B12)
$$\varrho =-\frac{1}{4\pi }\left\{\frac{1}{R}\frac{\partial }{\partial R}\left(R\frac{\partial }{\partial R}\right)+\frac{1}{R^{2}}\frac{\partial^{2}}{\partial \phi^{2}}+\frac{\partial^{2}}{\partial z^{2}}\right\}\varphi .$$

For ease of calculation, we can separate out each term of the total charge density, $\varrho =\varrho_{R}+\varrho_{z}+\varrho_{\phi }$. The R-component $\varrho_{R}$ is given by

(B13)
$$\varrho_{R}=\;-2a_{DNA}\overset{‾}{\sigma }\sum_{n=0}^{\infty }\;\frac{e^{-\frac{1}{2}n^{2}g^{2}\Delta_{eff}^{2}}}{\delta_{n,0}+1}cos[n(\phi -gz)]cos\left[\frac{n\phi_{s}}{2}\right]{𝒲}_{n}^{\left(2\right)}\left(R;a_{DNA},\kappa \right)\;\;+2a_{CC}\overset{‾}{\sigma }\Theta \sum_{n=0}^{\infty }\;\frac{\left(f_{1}e^{-\frac{1}{2}n^{2}g^{2}\delta z_{c1}^{2}}+f_{2}(-1{)}^{n}e^{-\frac{1}{2}n^{2}g^{2}\delta z_{c2}^{2}}\right)}{\delta_{n,0}+1}cos[n(\phi -gz)]{𝒲}_{n}^{\left(2\right)}\left(R;a_{CC},\kappa \right)\;\;+2a_{W}{\overset{‾}{P}}_{0}\sum_{n=0}^{\infty }\;\frac{\left(w_{1}e^{-\frac{1}{2}n^{2}g^{2}\delta z_{w1}^{2}}+w_{2}(-1{)}^{n}e^{-\frac{1}{2}n^{2}g^{2}\delta z_{w2}^{2}}\right)}{\delta_{n,0}+1}cos[n(\phi -gz)]{𝒲}_{n}^{\left(2\right)}\left(R;a_{W},\kappa \right),$$

where ${𝒲}_{n}^{\left(2\right)}=(1/R)\partial \left(R{𝒲}_{n}^{\prime }\right)/\partial R$. When writing the smeared charge density $\overset{‾}{Q}$, it is also convenient to separate ${𝒲}_{n}^{\left(2\right)}\left(R;a_{\nu },\kappa \right)$ into three terms:

(B14)
$${𝒲}_{n}^{\left(2\right)}={𝒲}_{n,R<a_{\nu }}^{\left(2\right)}+{𝒲}_{n,R=a_{\nu }}^{\left(2\right)}+{𝒲}_{n,R>a_{\nu }}^{\left(2\right)}.$$

Here, ${𝒲}_{n,R=a_{\nu }}^{\left(2\right)}=\delta \left(R-a_{\nu }\right)$; using the truncated Gaussian distribution defined in Eq. (44), the smearing of this term gives

(B15)
$$\overset{ˆ}{\zeta }{𝒲}_{n,R=a_{\nu }}^{\left(2\right)}=\sqrt{\frac{2}{\pi }}\frac{\Theta (R)}{\delta a_{\nu }\left(1+erf\left[\frac{{\overset{‾}{a}}_{\nu }}{\sqrt{2}\delta a_{\nu }}\right]\right)}exp\left[\;-\;\frac{{\left(R-{\overset{‾}{a}}_{\nu }\right)}^{2}}{2\delta a_{\nu }^{2}}\right].$$

The left and right terms, when smeared, are calculated numerically. We can also write expressions for the z and ϕ components,

(B16)
$$\varrho_{z}=2a_{DNA}\overset{‾}{\sigma }\sum_{n=1}^{\infty }\;n^{2}g^{2}e^{-\frac{1}{2}n^{2}g^{2}\Delta_{\text{eff}}^{2}}cos[n(\phi -gz)]cos\left[\frac{n\phi_{s}}{2}\right]{𝒲}_{n}\left(R;a_{DNA},\kappa \right)\;\;-2a_{CC}\overset{‾}{\sigma }\Theta \sum_{n=1}^{\infty }\;n^{2}g^{2}\left(f_{1}e^{-\frac{1}{2}n^{2}g^{2}\delta z_{c1}^{2}}+f_{2}(-1{)}^{n}e^{-\frac{1}{2}n^{2}g^{2}\delta z_{c2}^{2}}\right)cos[n(\phi -gz)]{𝒲}_{n}\left(R;a_{CC},\kappa \right)\;\;-2a_{W}{\overset{‾}{P}}_{0}\sum_{n=1}^{\infty }\;n^{2}g^{2}\left(w_{1}e^{-\frac{1}{2}n^{2}g^{2}\delta z_{w1}^{2}}+w_{2}(-1{)}^{n}e^{-\frac{1}{2}n^{2}g^{2}\delta z_{w2}^{2}}\right)cos[n(\phi -gz)]{𝒲}_{n}\left(R;a_{W},\kappa \right),$$

(B17)
$$\varrho_{\phi }=\frac{2a_{DNA}\overset{‾}{\sigma }}{R^{2}}\sum_{n=1}^{\infty }\;n^{2}e^{-\frac{1}{2}n^{2}g^{2}\Delta_{\text{eff}}^{2}}cos[n(\phi -gz)]cos\left[\frac{n\phi_{s}}{2}\right]{𝒲}_{n}\left(R;a_{DNA},\kappa \right)\;\;-\frac{2a_{CC}\overset{‾}{\sigma }\Theta }{R^{2}}\sum_{n=1}^{\infty }\;n^{2}\left(f_{1}e^{-\frac{1}{2}n^{2}g^{2}\delta z_{c1}^{2}}+f_{2}(-1{)}^{n}e^{-\frac{1}{2}n^{2}g^{2}\delta z_{c2}^{2}}\right)cos[n(\phi -gz)]{𝒲}_{n}\left(R;a_{CC},\kappa \right)\;\;-\frac{2a_{W}{\overset{‾}{P}}_{0}}{R^{2}}\sum_{n=1}^{\infty }\;n^{2}\left(w_{1}e^{-\frac{1}{2}n^{2}g^{2}\delta z_{w1}^{2}}+w_{2}(-1{)}^{n}e^{-\frac{1}{2}n^{2}g^{2}\delta z_{w2}^{2}}\right)cos[n(\phi -gz)]{𝒲}_{n}\left(R;a_{W},\kappa \right),$$

where smearing is applied in a way similar to the above. These expressions for the magnitude of the electric field and the charge density are plotted in Figs. 3(e) and 3(f).

## APPENDIX C: EFFECT OF STRUCTURAL FLUCTUATIONS ON THE ELECTROSTATIC PROPERTIES OF DNA IN SOLUTION

In the main text, we used MD simulations to determine the electrostatic properties of DNA. In those simulations, the DNA atoms were restrained to their initial coordinates using rather stiff harmonic potentials (spring constant $k_{\text{spring}}=10\;kcal{\;mol}^{-1\;}{\text{Å}}^{-2}$). In order to investigate the potential impact of structural fluctuations on our conclusions, we conducted two additional simulations using much weaker harmonic restraints (spring constant $k_{\text{spring}}=0.5\;kcal{\;mol}^{-1}{\;\text{Å}}^{-2}$) for the 0.154-MKCl and pure water electrolyte conditions.

While the cylindrically averaged 2D maps [Fig. 10(a)] appear quite similar to and visually indistinguishable from those with strong restraints on DNA atoms [Fig. 5(a)], the corresponding line plots [Figs. 10(b) and 10(c)] exhibit a slight lateral shift. The oscillatory peaks align well but are shifted by approximately 0.4 Å away from the center of the DNA helix. This shift is caused by the softer restraints on DNA atoms, resulting in an average RMSD of the non-hydrogen atoms of about 0.68 Å compared to 0.28 Å with the stronger restraints, yielding a relative difference of around 0.4 Å. A similar shift is observed in the difference of the radially averaged potentials obtained using fine and coarse electrostatic calculations [Fig. 10(c)], following the analysis described in Fig. 5(c). Similar oscillations are also seen when averaged with respect to the reference base pair [Figs. 10(d) and 10(e)].

Despite the similarity in the potential with and without strong restraints, the dipole moment pattern is significantly disrupted with the weaker restraints. In the KCl system, the dipole moment is nearly completely disordered [Fig. 10(f), top]. In contrast, the pure water system displays an asymmetric pattern (Fig. 10, bottom), with more order within the minor groove. This finding can be attributed to the higher tendency of water molecules to become trapped within the minor groove [27].

## APPENDIX D: DIRECT COMPARISON BETWEEN THEORY AND SIMULATIONS

The electrostatic potential as calculated in the theory is directly compared here against the results of the molecular dynamics simulations in Fig. 11.

Inside the DNA, for R<10Å, we can clearly see that, despite there being qualitative similarities, it is not possible to make quantitative comparisons between the results of the theory and simulation. This finding can be attributed to the theory not explicitly considering the contribution of the quadrupolar moment to the adsorbed water in the grooves. A preliminary analysis of the molecular dynamics simulations shows that this quadrupolar contribution is incredibly strong, leading to the massive values of potential inside the DNA relative to the bulk, in line with previous suggestions [61]. However, given the size of the simulated system, this analysis itself is limited by computational power and memory, so a more detailed investigation of this effect is left to future work. Regardless of quantitative differences, both the theory and simulations point towards structured water in the grooves as the main reason for this positive potential inside the DNA.

Outside the DNA, for R>10Å, a much better agreement is found. Not only do the theory and simulation match well in terms of absolute value of the potential but also in terms of its oscillating features. Of course, within about 2.5 Å of the phosphate group, there are some qualitative differences, which can be attributed to nonlinear response and dielectric saturation effects neglected within the linear response theory developed here, which of course will be present in the simulations. Such effects were discussed above in the context of the embedded charge approximation, and they have been explored in detail in Refs. [49,88].
